# The determinants of lipid profiles in early adolescence in a Ugandan birth cohort

**DOI:** 10.1038/s41598-021-96035-x

**Published:** 2021-08-13

**Authors:** Jan Pieter R. Koopman, Swaib A. Lule, Christopher Zziwa, Hellen Akurut, Lawrence Lubyayi, Margaret Nampijja, Florence Akello, Priscilla Balungi, Josephine Tumusiime, Gloria Oduru, Alison M. Elliott, Emily L. Webb, John Bradley

**Affiliations:** 1 Department of Infectious Disease Epidemiology, London School of Hygiene and Tropical Medicine, London, UK; 2 Department of Parasitology, Leiden University Medical Centre, Albinusdreef 2, 2333 ZA Leiden, The Netherlands; 3 Institute for Global Health, University College London, London, UK; 4 Immunomodulation and Vaccines Programme, MRC/UVRI & LSHTM Uganda Research Unit, Entebbe, Uganda; 5 Department of Clinical Research, London School of Hygiene and Tropical Medicine, London, UK; 6 African Population and Health Research Center, Nairobi, Kenya

**Keywords:** Dyslipidaemias, Risk factors, Epidemiology

## Abstract

Dyslipidaemia in adolescence tracks into adulthood and is an important risk factor for cardiovascular disease. Little is known about the effects of environmental exposures and early-life exposure to infectious diseases common to tropical regions on lipids. In 1119 early adolescent participants in the Entebbe Mother and Baby Study, we used linear regression to examine whether prenatal, childhood or adolescent factors are associated with lipid levels. Reduced high-density lipoprotein (HDL) and elevated triglyceride levels were common (prevalence 31% and 14%, respectively), but elevated low-density lipoprotein (LDL) or total cholesterol (TC) were rare. Current malaria infection was associated with lower mean LDL (adjusted ß − 0.51; 95% CI − 0.81, − 0.21), HDL (adjusted ß − 0.40; 95% CI − 0.56, − 0.23), and TC levels (adjusted ß − 0.62; 95% CI − 0.97, − 0.27), but higher mean triglyceride levels (geometric mean ratio (GMR) 1.47; 95% CI 1.18–1.84). Early-life asymptomatic malaria was associated with modest reductions in HDL and TC. Body mass index (BMI) was positively associated with LDL, TC, and triglycerides. No associations with helminth infection were found. Our findings suggest that early-life factors have only marginal effects on the lipid profile. Current malaria infection and BMI are strongly associated with lipids and important to consider when trying to improve the lipid profile.

## Introduction

Cardiovascular diseases (CVDs) are the leading cause of death in the world^1^, causing 31% of global deaths in 2016^2^. Nearly 75% of these deaths occurred in low- and middle-income countries (LMICs)^1^. The pathophysiology leading to the end stages of CVD is complex and many risk factors have been identified, with smoking, obesity, diabetes, hypertension and abnormal cholesterol levels implicated to be of particular importance^3–5^. Abnormal cholesterol levels, or dyslipidaemia, accelerate atherosclerosis, with detrimental effects on health^6^.

The prevalence of dyslipidaemia is high in several countries. In 2008, the World Health Organization (WHO) estimated the prevalence of dyslipidaemia at 50–60% in North America and > 60% in some Western European countries^7^. In Africa, dyslipidaemia is common in adults: a recent systematic review and meta-analysis reported a prevalence of 29% for elevated low-density lipoprotein (LDL) and 37% for reduced high-density lipoprotein (HDL)^8^. Interventions aimed at reducing LDL levels consist of drug therapy^9^ and lifestyle changes, such as healthier diet, smoking cessation and increased physical activity^10^. Improvement of HDL levels can be achieved through similar lifestyle changes^11^.

Dyslipidaemia is less common in adolescents, yet, if present, is likely to increase the risk of CVD events later in life and may lead to early death, because lipid levels in adolescence are strongly correlated with lipid levels in later life^12,13^. Epidemiological data from several cohort studies indicate that high LDL and low HDL in adolescents strongly predict high-risk carotid intima-media thickness (cIMT) in adults, which is used as a surrogate for early artherosclerosis^14^. Understanding which factors influence lipid levels, particularly LDL and HDL, in adolescents may therefore inform intervention strategies to reduce CVD risk.

Much of the research on risk factors for dyslipidaemia has focussed on anthropometrics and lifestyle factors. Studies have shown that increased body mass index (BMI) and body fat percentage are strongly associated with dyslipidaemia^15,16^. With regard to lifestyle factors, many studies have explored the effect of nutritional intake and physical activity on the lipid profile. Others have studied whether early-life factors influence lipid levels: exclusive breastfeeding in infancy, for instance, has been found to be associated with healthier lipid profiles in adolescence^17^. Evidence for an association between birthweight and later lipid concentration is so far inconclusive^18^.

Apart from birthweight and breastfeeding, little is known about the effect of other early-life factors on lipid concentrations later in life. In particular, studies examining the potential effects of environmental factors and early-life exposure to infectious diseases that are more common in LMICs are lacking. Some data have suggested that parasitic infections interfere with lipid metabolism and therefore may lead to changes in lipid profiles^19^; this phenomenon has been well described for malaria parasites^20^, but not for others. Moreover, childhood exposure to helminth infections has been shown to reduce the likelihood of developing immune-mediated disorders in later life, due to the immuno-modulatory effects of helminths, which skew the immune system towards a more immunoregulatory response^21^. This may be relevant as inflammation plays an important role in dyslipidaemia and atherosclerosis^22^. From this, we hypothesise that childhood exposure to parasites may have a beneficial effect on the lipid profile later in life.

Many African countries are experiencing a societal and epidemiological transition, and seeing rapid urbanisation together with the burden of disease shifting away from infectious diseases towards non-communicable diseases (NCDs)^8^. As a result of these changes, CVD is expected to become an even bigger health problem in the near future. Therefore, this study aimed to explore whether early-life or other life factors were associated with lipid levels in African adolescents using data from the Entebbe Mother and Baby Study (EMaBS) birth cohort in Uganda^23^. Specifically, we hypothesised that exposure to environmental factors and early-life exposure to infectious diseases that are common in this setting, might influence lipid levels. The results will help us better understand the relation between life-course factors and lipid levels in a setting where dyslipidaemia and CVDs are on the increase.

## Results

### Participant characteristics

From the 2345 mother and baby pairs in the EMaBS birth cohort, 1119 children (48%) participated in the blood pressure (BP) study within which lipid profiles were examined. For most characteristics, participants were similar to non-participants with the exception that the BP study participants were more likely to be HIV negative and to have a mother who had received higher education, was married, and did not have hookworm infection^24^.

The baseline characteristics of the study participants are shown in Table 1. Of the 1119 participants, 536 (48%) were girls. Their mean age at measurement of lipid levels was 10.4 years (SD 0.49; range 10.0–12.0) and mean BMI at time of lipid measurements was 16.0 kg/m^2^ (SD 1.88). Infections were frequently observed in childhood: Respectively, 58% and 11% of the adolescents experienced clinical malaria and at least one episode of asymptomatic malaria in childhood (below the age of 5 years). HIV prevalence in the children was 2% (vertically acquired from their mothers) and another 9% had been exposed to HIV (vertical exposure) without being infected. At the time of lipid measurement, *Schistosoma mansoni* was the most common infection found (10%), followed by *Trichuris* (4%), malaria (2%), and hookworm (1%). *Ascaris* was only diagnosed in three adolescents.

**Table 1:** Characteristics of study participants (N = 1119).

			N (%)	Mean (SD)
Level 1: Socioeconomic factors	Household SES (missing: n = 5)	Low	142 (12.9)	
		Middle	642 (58.1)	
		High	320 (29.0)	
	Maternal education (missing: n = 2)	None	28 (2.5)	
		Primary	542 (48.5)	
		Senior	438 (39.2)	
		Tertiary	109 (9.8)	
	Marital status (missing: n = 1)	Single	116 (10.4)	
		Married	967 (86.5)	
		Other	35 (3.1)	
	Area of residence (missing: n = 3)	Urban	770 (69.6)	
		Rural	336 (30.4)	
Level 2: Reproductive factors	Maternal age (years)	14–19	220 (19.7)	
		20–24	429 (38.3)	
		25–29	269 (24.0)	
		30 +	201 (18.0)	
	Maternal BMI (kg/m^2^) (missing: n = 9)			24.1 (3.37)
	Alcohol during pregnancy (missing: n = 1)	No	775 (69.3)	
		Yes	343 (30.7)	
(Maternal infection)	*Schistosoma mansoni *(missing: n = 7)	No	908 (81.7)	
		Yes	204 (18.3)	
(Maternal infection)	*Ascaris *(missing: n = 7)	No	1084 (97.5)	
		Yes	28 (2.5)	
(Maternal infection)	*Trichuris *(missing: n = 7)	No	1015 (91.3)	
		Yes	97 (8.7)	
(Maternal infection)	Hookworm (missing: n = 7)	No	662 (59.5)	
		Yes	450 (40.5)	
(Maternal infection)	Asymptomatic malaria (missing: n = 9)	No	991 (90.1)	
		Yes	109 (9.9)	
	Treatment allocation 1	Placebo	566 (50.6)	
		Albendazole	553 (49.4)	
	Treatment allocation 2	Placebo	564 (50.4)	
		Praziquantel	555 (49.6)	
Level 3: Perinatal factors	Sex	Female	536 (47.9)	
		Male	583 (52.1)	
	Low birthweight (missing: n = 187)	No	867 (93.0)	
		Yes	65 (7.0)	
	Mode of delivery (missing: n = 2)	Vaginal	1005 (90.0)	
		C-section/instrument	112 (10.0)	
	Place of delivery (missing n = 1)	Hospital	824 (73.7)	
		Home	120 (10.7)	
		Other	174 (15.6)	
	Season	Wet	568 (50.8)	
		Dry	551 (49.2)	
	Exclusively breastfed at 6 weeks (missing: n = 13)	No	358 (32.4)	
		Yes	748 (67.6)	
Level 4: Childhood infections	Treatment allocation 3 (missing n = 12)	Placebo	553 (50.0)	
		Albendazole	554 (50.0)	
(Infection age ≤ 5)	*Schistosoma mansoni *(missing: n = 10)	No	1076 (97.0)	
		Yes	33 (3.0)	
(Infection age ≤ 5)	*Ascaris *(missing: n = 10)	No	1052 (94.9)	
		Yes	57 (5.1)	
(Infection age ≤ 5)	*Trichuris *(missing: n = 10)	No	997 (89.9)	
		Yes	112 (10.1)	
(Infection age ≤ 5)	Hookworm (missing: n = 10)	No	1085 (97.8)	
		Yes	24 (2.2)	
(Infection age ≤ 5)	Asymptomatic malaria (missing: n = 12)	No	983 (88.8)	
		Yes	124 (11.2)	
(Infection age ≤ 5)	Clinical malaria	No	474 (42.4)	
		Yes	645 (57.6)	
(Infection age ≤ 5)	HIV exposure status	Unexposed	1001 (89.5)	
		Exposed, but uninfected	100 (8.9)	
		Infected	18 (1.6)	
Level 5: Lifestyle factors and current infections	Age (years)			10.4 (0.50)
	BMI (kg/m^2^)			16.0 (1.88)
	Tanner pubic hair growth stage (missing: n = 509)	Stage 1	441 (72%)	
		Stage 2	149 (24%)	
		Stage 3	20 (3%)	
	Tanner breast development stage, only in girls (missing: n = 262)	Stage 1	178 (65%)	
		Stage 2	79 (29%)	
		Stage 3	17 (6%)	
(Current infection)	*Schistosoma mansoni *(missing: n = 43)	No	964 (89.6)	
		Yes	112 (10.4)	
(Current infection)	*Trichuris *(missing: n = 43)	No	1036 (96.3)	
		Yes	40 (3.7)	
(Current infection)	Hookworm (missing: n = 43)	No	1066 (99.1)	
		Yes	10 (0.9)	
(Current infection)	Asymptomatic malaria (missing n = 30)	No	1067 (98.0)	
		Yes	22 (2.0)	
	Days of fruit eaten/week (missing: n = 35)	None	114 (10.5)	
		1–3	620 (57.2)	
		4–7	350 (32.3)	
	Days of vegetables eaten/week (missing: n = 33)	None	106 (9.7)	
		1–3	575 (52.5)	
		4–7	415 (37.8)	
	Days of animal-protein eaten/week (missing: n = 9)	None	122 (11.1)	
		1–3	786 (71.4)	
		4–7	192 (17.5)	
	Days of sugared drinks taken/week (missing: n = 26)	None	427 (39.1)	
		1–3	492 (45.0)	
		4–7	174 (15.9)	
	Physical education at school (missing: n = 15)	No	385 (34.9)	
		Yes	719 (65.1)	

**Figure 1:**
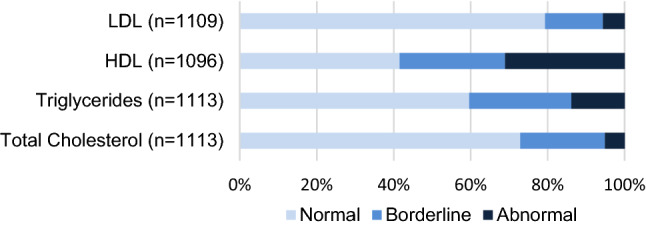
Distribution of lipid levels into categories according to AHA guidelines.

Serum LDL, HDL, and TC levels were successfully measured in 1109, 1096, and 1113 individuals, respectively; mean levels were 2.28 mmol/L (SD 0.66), 1.14 mmol/L (SD 0.34), and 3.82 mmol/L (SD 0.78). Triglycerides were measured in 1113 individuals (geometric mean: 0.81 mmol/L; SD 0.44). Figure 1 shows the distribution of these lipids when categorised into normal, borderline, and abnormal levels according to the American Heart Assosciation reference values for children^25^. Abnormal levels of HDL and triglycerides were observed in 31% and 14% of the adolescents, respectively. Only 5% of the adolescents had abnormal LDL levels and 5% had abnormal TC levels.

### Factors affecting LDL levels

Supplementary Table S1 shows the relationship with LDL levels for all variables considered, while Table 2 shows variables with evidence of association with LDL and that were retained for model building. Socioeconomic variables only appeared to be weakly associated with LDL. Among the reproductive and perinatal factors, no evidence was found for associations with LDL, after adjustment for counfounders. Of the infections in childhood, there was evidence for an association between LDL and *Trichuris* infection with higher LDL values in those infected with *trichuris* below the age of five (adjusted ß 0.15; 95% CI 0.02, 0.29; p = 0.03). Other childhood infections were not associated with LDL. BMI at time of lipid measurement was linearly associated with LDL levels (adjusted ß 0.03; 95% CI 0.01, 0.06; p = 0.006). Lower LDL levels were observed in those with malaria infection at time of lipid measurement (adjusted ß − 0.51; 95% CI − 0.81, − 0.21, p = 0.001). In the adjusted analysis, increased frequency of animal protein consumption and reduced frequency of fruit intake were both associated with higher LDL levels. Physical education (PE) at school did not appear to be associated with LDL.

**Table 2:** Hierarchical multiple linear regression of risk factors for LDL.

		Crude change in LDL (mmol/L) for every one-unit increase of the exposure variable from baseline (95% CI)	p value	Adjusted change in LDL (mmol/L) for every one-unit increase of the exposure variable from baseline (95% CI)	p value
Model 1 (n = 1080)	**Household SES**				
	Low	Reference	0.09	Reference	0.09
	Middle	− 0.05 (− 0.17, 0.07)		− 0.08 (− 0.20, 0.05)	
	High	0.05 (− 0.09, 0.18)		0.02 (− 0.11, 0.16)	
	**Marital status**				
	Single	− 0.05 (− 0.17, 0.08)	0.03	− 0.07 (− 0.20, 0.06)	0.06
	Married	Reference		Reference	
	Other^a^	− 0.30 (− 0.52, − 0.08)		− 0.26 (− 0.50, 0.02)	
Model 2 (n = 1061)	Maternal BMI (kg/m^2^)	0.01 (0.00–0.03)	0.01	0.01 (0.00, 0.02)	0.08
Model 3 (n = 890)	**Sex**				
	Female	Reference	0.008	Reference	0.10
	Male	− 0.11 (− 0.18, − 0.03)		− 0.07 (− 0.16, 0.01)	
Model 4 (n = 1063)	***Trichuris* below the age of 5**				
	No	Reference	0.05	Reference	0.03
	Yes	0.13 (0.00, 0.26)		0.15 (0.02, 0.29)	
	**Asymptomatic malaria before the age of 5**				
	No	Reference	0.02	Reference	0.10
	Yes	− 0.15 (− 0.27, − 0.02)		− 0.11 (− 0.24, 0.02)	
Model 5 (n = 954)	BMI (kg/m^2^)	0.04 (0.02, 0.06)	< 0.001	0.03 (0.01, 0.06)	0.006
	**Current asymptomatic malaria infection**				
	No	Reference	< 0.001	Reference	0.001
	Yes	− 0.53 (− 0.82, − 0.24)		− 0.51 (− 0.81, − 0.21)	
	**Days of fruit eaten/week**				
	None	Reference	0.53	Reference	0.03
	1–3	− 0.08 (− 0.21, 0.06)		− 0.20 (− 0.35, − 0.05)	
	4–7	− 0.07 (− 0.21, 0.07)		− 0.14 (− 0.30, 0.03)	
	**Days of animal protein eaten/week**				
	None	Reference	0.01	Reference	0.002
	1–3	0.06 (− 0.06, 0.19)		0.10 (− 0.04, 0.24)	
	4–7	0.20 (0.05, 0.35		0.27 (0.11, 0.44)	

Model 1: Age, sex + other variables at level 1.

Model 2: Age, sex, household SES, marital status + other variables at level 2.

Model 3: Age, sex, household SES, marital status, maternal BMI + other variables at level 3.

Model 4: Age, sex, household SES, marital status, maternal BMI + other variables at level 4.

Model 5: Age, sex, household SES, marital status, maternal BMI, *trichuris* below age of 5, malaria below age of 5 + other variables at level 5.

^a^Other: widowed, divorced, or separated.

### Factors affecting HDL levels

Supplementary Table S2 shows the relationship with HDL levels for all variables considered, while Table 3 shows variables with evidence of association with HDL and that were retained for model building. At the most distal level, the lowest HDL levels were observed in children with low household SES and in those whose mothers had not been schooled. On the second level, some evidence for an association between maternal age and HDL was found. From the maternal infections, some evidence was observed for an association between hookworm and HDL (adjusted ß − 0.06; 95% CI − 0.10, − 0.01; p = 0.01). HDL levels in children born with low birthweight were lower than those born with normal weight. Children born in dry season had slightly lower HDL levels than those born in wet season and children born by caesarean section or with the help of instruments had slightly higher HDL levels than those born vaginally. Of the infections during childhood, children who had at least one episode of asymptomatic malaria showed lower HDL levels (adjusted ß − 0.04; 95% CI − 0.15, 0.00, p = 0.04). No evidence for an association between childhood clinical malaria and HDL was observed. At the level of lifestyle factors and current infections, comparable to LDL, current malaria infection was associated with a large reduction in HDL levels (adjusted ß − 0.40; 95% CI − 0.56, − 0.23; p < 0.001). Children who drank sugared drinks more frequently had higher HDL values. No evidence for an association between PE at school and HDL was observed.

**Table 3:** Hierarchical multiple linear regression of risk factors for HDL.

		Crude change in HDL (mmol/L) for every one-unit increase of the exposure variable from baseline (95% CI)	p value	Adjusted change in HDL (mmol/L) for every one-unit increase of the exposure variable from baseline (95% CI)	p value
Model 1 (n = 1068)	**Household SES**				
	Low	Reference	< 0.001	Reference	< 0.001
	Middle	0.06 (0.00, 0.12)		0.05 (− 0.01, 0.12)	
	High	0.14 (0.07, 0.21)		0.13 (0.05, 0.19)	
	**Maternal education**				
	None	− 0.19 (− 0.31, − 0.06)	0.005	− 0.17 (− 0.30, − 0.04)	0.03
	Primary	Reference		Reference	
	Senior	0.04 (− 0.01, 0.08)		0.03 (− 0.02, 0.07)	
	Tertiary	0.03 (− 0.03, 0.10)		0.02 (− 0.06, 0.09)	
Model 2 (n = 1046)	**Maternal age (years)**				
	14–19	Reference	0.03	Reference	0.04
	20–24	− 0.05 (− 0.11, 0.00)		− 0.04 (− 0.10, 0.02)	
	25–29	− 0.05 (− 0.11, 0.01)		− 0.03 (− 0.11, 0.05)	
	30+	0.02 (− 0.05, 0.09)		0.05 (− 0.05, 0.14)	
	**Maternal hookworm infection**				
	No	Reference	< 0.001	Reference	0.01
	Yes	− 0.07 (− 0.12, − 0.03)		− 0.06 (− 0.10, − 0.01)	
	**Treatment allocation of mother to praziquantel**				
	No	Reference	0.08	Reference	0.08
	Yes	− 0.04 (− 0.08, 0.01)		− 0.04 (− 0.08, 0.00)	
Model 3 (n = 882)	**Mode of delivery**				
	Vaginal	Reference	0.004	Reference	0.01
	C-section/instrument	0.10 (0.03, 0.17)		0.10 (0.02, 0.17)	
	**Season at birth**				
	Wet	Reference	0.03	Reference	0.007
	Dry	− 0.04 (− 0.08, 0.00)		− 0.06 (− 0.11, − 0.02)	
	**Low birthweight**				
	No	Reference	0.01	Reference	0.04
	Yes	− 0.11(− 0.19. − 0.03)		− 0.08 (− 0.15, 0.00)	
Model 4 (n = 875)	**Asymptomatic malaria below the age of 5**				
	No	Reference	0.005	Reference	0.04
	Yes	− 0.09 (− 0.16, − 0.03)		− 0.08 (− 0.16, − 0.01)	
Model 5 (n = 784)	**Current asymptomatic malaria infection**				
	No	Reference	< 0.001	Reference	< 0.001
	Yes	− 0.49 (− 0.64, − 0.35)		− 0.40 (− 0.56, − 0.23)	
	**Days of vegetables eaten/week**				
	None	Reference	0.98	Reference	0.09
	1–3	0.00 (− 0.07, 0.07)		− 0.09 (− 0.18, 0.00)	
	4–7	0.00 (− 0.07, 0.07)		− 0.09 (− 0.18, 0.04)	
	**Days of sugary drinks taken/week**				
	None	Reference	< 0.001	Reference	< 0.001
	1–3	0.02 (− 0.03, 0.06)		0.01 (− 0.04, 0.07)	
	4–7	0.13 (0.07, 0.19)		0.13 (0.06, 0.20)	

Model 1: Age, sex + other variables at level 1.

Model 2: Age, sex, household SES, maternal education + other variables at level 2.

Model 3: Age, sex, household SES, maternal education, maternal age, maternal hookworm infection, treatment allocation to praziquantel + other variables at level 3.

Model 4: Age, sex, household SES, maternal education, maternal age, maternal hookworm infection, treatment allocation to praziquantel, low birthweight, mode of delivery, season + other variables at level 4.

Model 5: Age, sex, household SES, maternal education, maternal age, maternal hookworm infection, treatment allocation to praziquantel, low birthweight, mode of delivery, season, malaria infection below the age of 5 + other variables at level 5.

### Factors affecting total cholesterol levels

Supplementary Table S3 shows the relationship with TC levels for all variables considered, while Table 4 shows variables with evidence of association with TC and that were retained for model building. On the most distal level, the highest TC levels were found in children in the high SES category and the lowest in middle SES. Among the reproductive and perinatal factors, we found only weak associations with maternal age and maternal malaria infection. Children born in the dry season had lower TC values than those born during the wet season. No evidence for associations was found for clinical malaria or other infections in childhood. Of the fifth level, several exposure variables were associated with TC. Firstly, there was evidence of a linear association between BMI and TC (p = 0.001). The adjusted mean difference in TC per one-unit increase of BMI was 0.05 mmol/L (95% CI 0.02, 0.08), which is similar to the estimate for LDL. Also, current malaria infection was associated with lower levels of TC (adjusted β − 0.62; 95% CI − 0.97, − 0.27 p < 0.001). The size and direction of the association is similar to that of LDL and HDL. From the dietary variables, increased intake of animal protein and reduced consumption of fruits were associated with higher TC.

**Table 4:** Hierarchical multiple linear regression of risk factors for total cholesterol.

		Crude change in TC (mmol/L) for every one-unit increase of the exposure variable from baseline (95% CI)	p value	Adjusted change in TC (mmol/L) for every one-unit increase of the exposure variable from baseline (95% CI)	p value
Model 1 (n = 1098)	**Household SES**				
	Low	Reference	0.02	Reference	0.03
	Middle	− 0.01 (− 0.15, 0.13)		− 0.04 (− 0.19, − 0.11)	
	High	0.14 (− 0.01, 0.30)		0.10 (− 0.05, 0.26)	
	**Marital status**				
	Single	0.01 (− 0.14, 0.16)	0.02	− 0.01 (− 0.16, 0.14)	0.08
	Married	Reference		Reference	
	Other^a^	− 0.39 (− 0.65, − 0.12)		− 0.32 (− 0.61, − 0.04)	
Model 2 (n = 1047)	**Maternal age group**				
	14–19	Reference	0.12	Reference	0.08
	20–24	− 0.04 (− 0.17, 0.09)		0.01 (− 0.13, 0.16)	
	25–29	0.11 (− 0.03, 0.25)		0.19 (0.01, 0.37)	
	30 +	0.03 (− 0.12, 0.18)		0.14 (− 0.07, 0.35)	
	**Maternal malaria infection**				
	No	Reference	0.05	Reference	0.08
	Yes	− 0.16 (− 0.31, 0.00)		− 0.15 (− 0.31, − 0.02)	
Model 3 (n = 885)	**Sex**				
	Female	Reference	0.005	Reference	0.08
	Male	− 0.13 (− 0.22, 0.04)		− 0.09 (− 0.20, 0.01)	
	**Season**				
	Wet	Reference	0.24	Reference	0.04
	Dry	− 0.06 (− 0.15, 0.04)		− 0.11 (− 0.21, 0.00)	
Model 4 (n = 1058)	**Asymptomatic malaria below the age of 5**				
	No	Reference	0.02	Reference	0.07
	Yes	− 0.17 (− 0.32, − 0.02)		− 0.14 (− 0.30, 0.01)	
Model 5 (n = 952)	BMI (kg/m^2^)	0.05 (0.03, 0.08)	< 0.001	0.04 (0.02, 0.08)	0.001
	**Current asymptomatic malaria infection**				
	No	Reference	< 0.001	Reference	< 0.001
	Yes	− 0.70 (− 1.04, − 0.37)		− 0.62 (− 0.97, − 0.27)	
	**Days of fruit eaten/week**				
	None	Reference	0.43	Reference	0.07
	1–3	− 0.07 (− 0.23, 0.08)		− 0.21 (− 0.39, − 0.03)	
	4–7	− 0.11 (− 0.28, 0.06)		− 0.19 (− 0.38, 0.00)	
	**Days of animal protein eaten/week**				
	None	Reference	0.02	Reference	0.001
	1–3	0.08 (− 0.07, 0.23)		0.11 (− 0.06, 0.27)	
	4–7	0.23 (0.05, 0.41)		0.33 (0.13, 0.53)	

Model 1: Age, sex + other variables at level 1.

Model 2: Age, sex, household SES, marital status + other variables at level 2.

Model 3: Age, sex, household SES, marital status, maternal age, maternal malaria infection + other variables at level 3.

Model 4: Age, sex, household SES, marital status, maternal age, maternal malaria infection, season + other variables at level 4.

Model 5: Age, sex, household SES, marital status, maternal age, maternal malaria infection, season, malaria infection below the age of 5 + other variables at level 5.

*TC* total cholesterol.

^a^Other: widowed, divorced, or separated.

### Factors affecting triglyceride levels

Supplementary Table S4 shows the relationship with triglyceride levels for all variables considered, while Table 5 shows variables with evidence of association with triglycerides and that were retained for model building. In contrast with the other lipids, no associations were found with socioeconomic variables. On the second level, some evidence was observed that these levels were associated with maternal age (p = 0.01), however a clear pattern or a similar trend with HDL and TC was lacking. There was some evidence that maternal hookworm was associated with increased triglyceride levels (adjusted GMR 1.05; 95% CI 0.99, 1.11; p = 0.08). Weak evidence for associations with place of delivery and low birthweight were also observed. Triglyceride levels in children born with low birthweight were 12% higher than in those born with normal weight (95% CI 1.00, 1.25; p = 0.05). No association was seen for asymptomatic malaria below the age of five. Triglyceride levels were slightly higher in children who were randomised to albendazole. On the most proximal level, in the adjusted analysis, triglyceride levels were estimated to increase by 3% (95% CI 1.01, 1.05) per one unit increase in BMI (p < 0.001). Also, in contrast with all other lipids, triglyceride levels were higher in those with current malaria infection (adjusted GMR 1.47; 95% CI 1.18, 1.84; p < 0.001). No associations were found between any of the dietary variables or physical activity level and triglyceride levels.

**Table 5:** Hierarchical multiple linear regression of risk factors for triglyceride levels.

		Crude geometric mean ratio (95% CI)	P value	Adjusted geometric mean ratio (95% CI)	P value
Model 2 (n = 1077)	**Maternal age group**				
	14–19	Reference	0.08	Reference	0.01
	20–24	1.03 (0.98, 1.11)		1.04 (0.96, 1.12)	
	25–29	1.10 (1.02, 1.19)		1.10 (1.00, 1.22)	
	30 +	0.97 (0.89, 1.05)		0.97 (0.86, 1.09)	
	**Maternal hookworm infection**				
	No	Reference	0.07	Reference	0.08
	Yes	1.05 (1.00, 1.11)		1.05 (0.99, 1.11)	
Model 3 (n = 912)	**Low birthweight**				
	No	Reference	0.07	Reference	0.05
	Yes	1.11 (1.00, 1.24)		1.12 (1.00, 1.25)	
	**Place of delivery**				
	Hospital	Reference	0.09	Reference	0.06
	Home	1.01 (0.93, 1.10)		0.91 (0.76, 1.08)	
	Other	1.08 (1.01, 1.16)		1.10 (1.00, 1.21)	
Model 4 (n = 905)	**Treatment allocation of child to albendazole**				
	No	Reference	0.08	Reference	0.05
	Yes	1.05 (1.00, 1.12)		1.06 (1.00, 1.12)	
Model 5 (n = 807)	BMI (kg/m^2^)	1.02 (1.01, 1.04)	< 0.001	1.03 (1.01, 1.05)	< 0.001
	**Current asymptomatic malaria infection**				
	No	Reference	< 0.001	Reference	0.001
	Yes	1.49 (1.24, 1.80)		1.47 (1.18, 1.84)	

Model 2: Age, sex + other variables at level 2.

Model 3: Age, sex, maternal age, maternal hookworm infection + other variables at level 3.

Model 4: Age, sex, maternal age, maternal hookworm infection, low birthweight, place of delivery + other variables at level 4.

Model 5: Age, sex, maternal age, maternal hookworm infection, low birthweight, place of delivery, treatment allocation to albendazole (child) + other variables at level 5.

## Discussion

This study aimed to investigate whether early-life or later exposures were associated with lipid levels in Ugandan, early adolescents. In the study cohort, abnormally low levels of HDL were observed in 31% of the adolescents, elevated triglyceride levels were seen in 14% and abnormally high levels of LDL and TC were found in around 5%. Although some associations were observed between variables at the more distal levels (variables related to socioeconomic status and reproductive factors) and lipid levels, the strongest associations were found at the most proximal level (variables related to lifestyle and current malaria infection). Our results show that current malaria infection is strongly associated with lipid levels. Adolescents with current malaria had lower LDL, HDL, and TC levels, but higher triglyceride levels. We also found some suggestion that childhood asymptomatic malaria infection, but not clinical malaria, was associated with decreased LDL, HDL and TC levels in adolescence. Current helminth infections and childhood exposure to helminths were not associated with lipid levels in adolescence. We found some evidence suggesting a relationship between dietary intake, e.g. animal protein consumption, and specific lipid levels. Also, our data showed that BMI was linearly positively associated with LDL, TC, and triglycerides.

The prevalence of dyslipidaemia in Ugandan adolescents was similar to that found in other LMICs including Brazil^26^, Ghana^27^, and Sri Lanka^28^; studies in these countries have all reported abnormally low HDL levels in around 30% of the participants. These abnormal HDL prevalence estimates are higher than those observed in adolescent populations in high-income countries such as United States^29^ (12.8%) or Korea^30^ (7.1%), and only slightly lower than those observed in the general adult African population^8^. Moreover, we observed low prevalence of elevated LDL levels in our study in contrast with what has been reported in adults^8^. Reference values from the American Health Association were used which made results comparable to other studies. However, these cut-offs may be less suitable for Ugandan adolesecents as serum HDL levels vary per population^31^ and as such further population studies will help define the population appropriate cut-offs for predicting cardiovascular risk. In addition, inclusion of other markers such as apoliprotein may be considered in future studies. Nevertheless, the high prevalence of decreased HDL remains worrying as HDL has a protective effect on the development of CVD^32^.

Our finding that current malaria infection affects the lipid profile is consistent with previous research. Visser et al. reported in their meta-analysis that decreased levels of LDL, HDL, and TC and increased levels of triglycerides levels were observed in malaria patients compared to healthy controls^20^, however in many of the studies included there was no appropriate controlling for confounders. We showed that after adjusting for a large number of potential confounders, such as early-life factors and coinfections, there remains strong evidence for an association between malaria infection and lipid levels. The effect estimates in our study are similar to those reported in this large meta-analysis^20^. Although the prevalence of current asymptomatic malaria infection was low, the effect size was large and potentially clinically relevant. Decreased HDL, LDL, and TC, but increased triglycerides are recognised in the acute phase response to infections^33^. However, malaria parasites may also interfere with cholesterol metabolism, which takes place in the liver, to ensure sufficient amounts are available for their survival^20^. Follow up of lipid levels after adequate malaria treatment may shed led on whether these changes are temporary or persist following infection clearance as this may have consequences for lipid improvement strategies. Another interesting question to explore is if lower HDL and LDL increase the likelihood of malaria infection.

Unlike for malaria, we did not find evidence for an association between current helminth infection and lipid levels. We did not find associations for *Schistosoma mansoni* infection which has been associated with lower LDL and TC in Ugandan adults^34^. However, the prevalence of these infections was low and the study was likely to be underpowered to find relevant differences. Our results are in agreement with data from Indonesia, where researchers did not find a difference in lipids between helminth-infected and helminth-uninfected adults after adjusting for sex, age, and BMI^35^.

To our knowledge, no previous studies have explored the effects of early-life exposure to helminths and malaria on lipid levels later in life. Although no evidence was observed for an association between lipids and helminths, the results suggest that asymptomatic malaria infection, but not clinical malaria, below the age of five may have a marginal effect on lipids. The directions of effects were the same as those for current malaria infection. The differences observed between childhood asymptomatic and clinical malaria with regard to lipids may be related to host immune responses. These responses in asymptomatic malaria are mainly anti-inflammatory^36^ and could, if this anti-inflamatory immune profile persists because treatment is not sought, potentially have some effect on lipid metabolism^37^.

We did not find evidence for an association between breastfeeding practices and lipid levels in contrast to a previous study in Hong Kong^17^. An explanation for this may be that the high-income setting of Hong Kong is very different to that in Uganda: the prevalence of abnormal HDL levels and the proportion of children being exclusively breastfed were much lower in the Hong Kong study. Also, dietary practices are likely to differ between Hong Kong and Uganda. Regarding low birthweight, we found some evidence suggesting that low birthweight was associated with lower HDL and higher triglyceride levels. The effect sizes however were small and the estimates may have been confounded as we could not control for gestational age.

Lifestyle factors were not the primary focus of this study. However, we did observe associations that have previously been described in other studies. The literature on the effects of dietary intake and lipid level is extensive and studies suggest that a healthy diet consisting of sufficient fruit and vegetable intake and restricted consumption of animal protein has a beneficial effect on the lipid levels^27^. In our study, we found similar results for some of the dietary variables. Our results for HDL showed a different pattern than what we expected, namely a positive association with consumption of sugary drinks. This is in contrast with cross-sectional studies in American children where no association was reported^38,39^. This finding may be a false positive result, but warrants further investigation. Moreover, we observed strong evidence for linear association of BMI with LDL, TC, and triglycerides. We did not find evidence for an association between physical education at school and lipids. It is important to note that the data on dietary intake and physical activity were self-reported and may not have been entirely accurate. Moreover, more detailed information, e.g. minutes of physical activity per day/type of activity, on these factors was lacking.

This study had several limitations. Although most of the characteristics of the participants were similar to non-participants, selection bias cannot be entirely ruled out. Also, data were missing for some variables, and because of the regression methods used, observations with missing data were excluded. For most variables, except for birthweight and puberty staging, the amount of missing data was small. Since birthweight was not the main exposure under investigation, we did not perform sensitivity analyses. Unfortunately, information on puberty staging was unavailable for nearly half of the participants, and therefore it was not included in the analyses.

Misclassification of some of the exposure variables may have occurred, for example through misdiagnosis of the infections and reporter bias in the survey data. In children with asymptomatic malaria and previous exposure to malaria, infections may be submicroscopic and therefore only detectable with molecular techniques^40^ and may have been underreported in our study. In this case, false negatives may have led to overestimation of the true effect. Similarly, for *Schistosoma mansoni* and hookworm infection the sensitivity of microscopy (using Kato-Katz) correlates with the infection intensity—light intensity infections are more likely to be missed^41^. The use of more novel antigen or molecular parasite detection methods in addition to microscopy (the gold standard) may be considered for future studies. Although we were able to control for many of the known confounders, data on some may have been missing and may have resulted in residual confounding. An example would be certain genetic traits that are related to lipid metabolism. Another potential limitation is the multiplicity of statistical tests that may have introduced spurious associations. When a variable appeared to be associated with more than one lipid, we considered this a stronger indication of a true association. Other factors that helped decide whether associations were not just due to chance were effect size, dose response, biological plausibility, and consistency with previously published literature. Due to the modelling strategy, the model assessing the effects of variables on the most proximal level (lifestyle and current infections) included many variables which may have increased the risk of overfitting. Lastly, for lifestyle factors and current infections the direction of the effects are difficult to establish because of the cross-sectional nature of these particular associations, while for the variables on the other levels this was not an issue.

The study included a large number of participants who where followed up for a long period of time making the results of this study generalisable to other adolescent populations in Sub-Saharan Africa with a similar parasitic burden. The longitudinal design of the study allowed us to explore the potential effects of early-life factors on adolescent lipid profiles in a setting where environmental and infectious disease exposure are different to those in high-income settings. To better understand if these exposures are associated with lipid levels is important, because Uganda, like many other LMICs, is experiencing societal changes and an epidemiological transition that will result in an increased burden of non-communicable diseases. Our high prevalence of reduced HDL levels shows the necessity of interventions aimed at improving lipid profiles to prevent the excess risk of CVD in adulthood. These interventions should focus on increasing HDL levels to healthier levels in order to avoid the development of artherosclerosis in early adulthood^14^ The implementation, however, may be challenging. For instance, we observed low HDL levels in adolescents with low household SES who may have limited access to healthy, yet affordable foods which may also depend on the area of residence. Interventions need to pay special attention to these different (socioeconomic) factors.

In conclusion, the prevalence of dyslipidaemia in Ugandan adolescents was high. Our findings suggest that early-life factors only have a marginal effect on the lipid profile. Current malaria infection and BMI are strongly associated with lipids and important to consider when trying to improve the lipid profile in adolescents to prevent excess risk of CVD in adulthood.

## Methods

### The EMaBS study

The Entebbe Mother and Baby Study (EMaBS) began as a randomised controlled trial (ISRCTN32849447) investigating the effect of deworming during pregnancy and early childhood on the infant’s immune response to immunisation, and on the susceptibility to infectious and allergy-related diseases^23^. The study was conducted in and around Entebbe, Uganda; the study setting comprised urban, peri-urban, rural and fishing communities on a peninsular in Lake Victoria A total of 2507 pregnant women recruited between April 2003 and November 2005. Using a 2 × 2 (× 2) factorial design, pregnant women were randomised to receive praziquantel or placebo and albendazole or placebo during pregnancy, and the resulting 2345 offspring were randomised to receive quarterly albendazole or placebo starting at 15 months to 5 years of age. All participants gave written, informed consent. This study was approved by ethics committees of the Uganda Virus Research Institute, the London School of Hygiene and Tropical Medicine and the Uganda National Council for Science and Technology and conducted in accordance with ICH-GCP guidelines and the Declaration of Helsinki. Around 31% of the eligible children in the community were enrolled into the EMaBS study. Participants were more likely to have higher SES and a lower risk of exposure to parasitic infection when compared to other children in the community^20^. At trial completion, children aged 5 years of age in this cohort continued under follow up (without intervention). Data were collected at scheduled visits starting in antenatal care, through delivery to childhood. Children also attended the clinic when ill, and their clinical data was recorded. Children still in the EMaBS birth cohort attending their 10 or 11 year routine annual visit between May 2014 and June 2016 were invited to participate in the blood pressure (BP) study. For those presenting at their annual visit with an illness, enrolment was postponed untill after the illness resolved. The BP study aimed to investigate influence of early-life, life-course and genetic factors on BP in adolescence. Additional written, informed consent was obtained from both the adolescent and their parent or guardian for participation in the BP study. Detailed study design and findings from the BP study have been published elsewhere^24,42,43^. In addition to BP measurements, Tanner staging for pubic hair and breast development, and anthropometric measurements for calculation of BMI , the 10- and 11-year old adolescents had blood samples collected for fasting lipid profile assessement. The lipid profiles were measured at the Uganda Virus Research Institute/Medical Research Council (UVRI/MRC) lab facility using the homogenous enzymatic colorimetric assay using Cobas Integra 400 plus (serial number 398794, Roche diagnostics Ltd). Total cholesterol (TC) was measured by enzymatic colorimetric method, which is standardised against the Isotope Dilution Mass Spectrometry (ID-Ms) and Abell Kendall. The assay’s lowest limit of detection is 0.1 mmol/L with an intermediate assay coefficient of variation (CV) % 1.6. Triglycerides were measured by enzymatic colorimetric principle, standardised against Isotope Dilution Mass Spectrometry, with its lowest limit of detection 0.1 mmol/L, intermediate assay CV% 1.5, LDL was measured by enzymatic colorimetric method which is standardised against beta quantification method,with its lowest limit of detection at 0.1 mmol/L,intermediate CV% 2.0, HDL was measured by enzymatic colorimetric method which standardised against ultra centrification method (CDC reference method), lowest limit of detection at 0.08 mmol/L and intermediate CV% 1.8 (cobas method sheets 2017-01 v7.0).

Blood samples were also examined for malaria parasitaemia using Leishman’s stains while stool was examined for helminth (*Schistosoma mansoni, Necator americanus, Ascaris lumbricoide*s and *Trichuris trichiura*) ova using Kato-Katz. Data on dietary intake and physical activity were obtained through a questionnaire completed through interviewing the adolescent with help of the parent/guardian. Participants were asked for how many days/week a given food/drink was consumed in a typical week of the last month. The foods and drinks surveyed included fruits, vegetables, sugared drinks, and animal-protein. Physical activity was surveyed by asking whether they were offered physical education at school and if yes, whether they participated^44^. Childhood HIV status was determined at 18 months of age using a rapid serial testing algorithm described elsewhere^45,46^. Data obtained in the BP study were linked to data previously collected in the EMaBS, for which detailed methods have been described elsewhere^45–47^. Using the data collected in the EMaBS, we aimed to investigate whether early-life and life-course factors affect blood lipid levels in early adolescence.

### Conceptual framework

**Figure 2:**
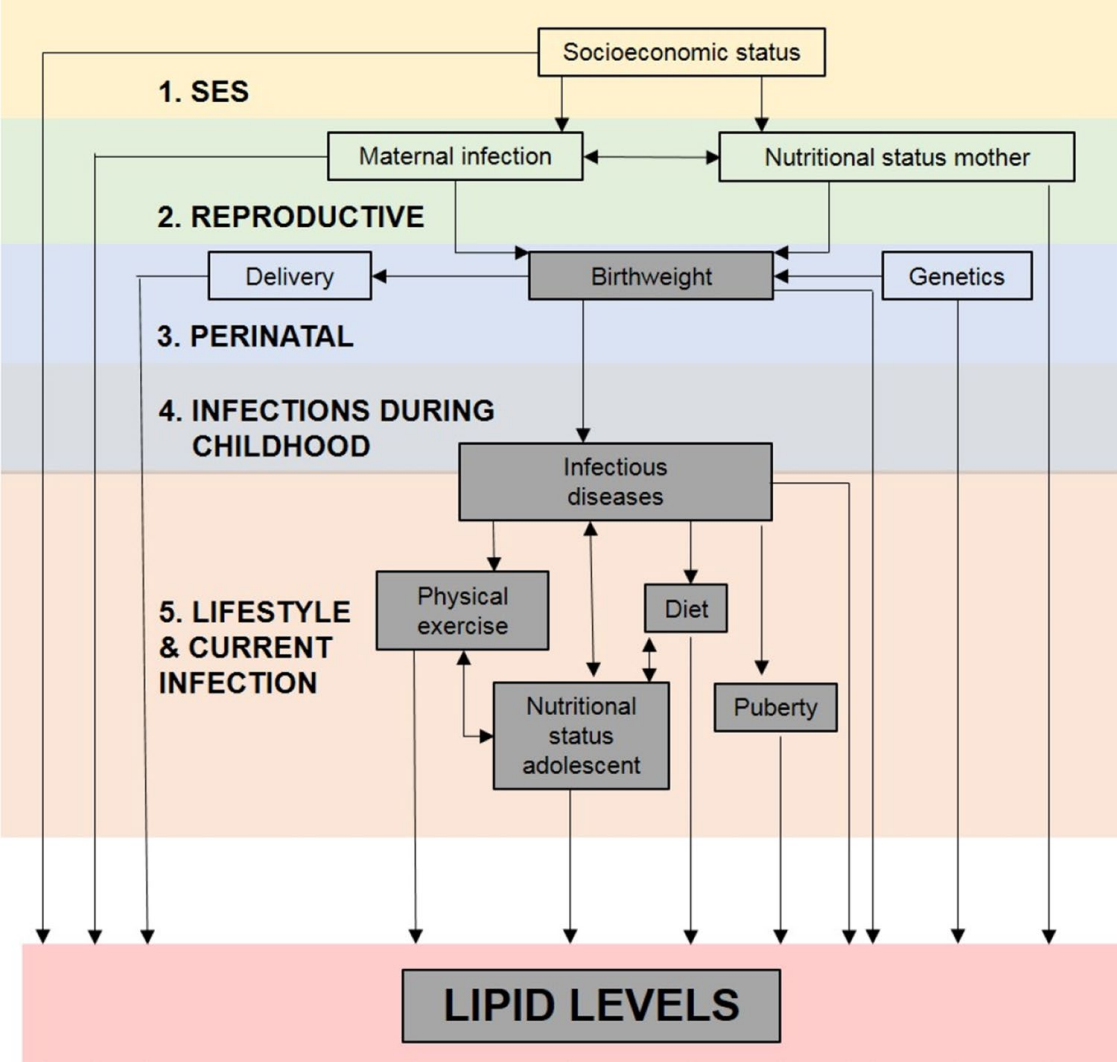
Proposed hierarchical conceptual framework for relationship between risk factors and lipid levels in adolescents. The boxes with grey shading indicate that these factors also depend on sex and age. The arrows represent the hypothesised relationships between the factors and can be bi-directional.

We hypothesised that lipid levels in adolescents might be affected by several risk factors at different stages in life, including socioeconomic factors, reproductive factors, perinatal factors, infections during childhood, infections at time of lipid measurement and lifestyle factors. To show the relationships between these factors a hierarchical conceptual framework was proposed (Fig. 2). According to this framework, socioeconomic status (SES) may either directly or indirectly determine all other variables, apart from sex and age at lipid measurement. The second level consisted of variables related to reproductive factors and included maternal nutritional status and maternal infections. These factors may partly have been determined by SES. The third level comprised of perinatal factors, e.g. birthweight, that may have been dependent on both SES and reproductive factors. Level 4 included variables related to childhood infections up to the age of 5 that may have been in part affected by SES, reproductive, and perinatal factors. Lastly, the most proximal level consisted of lifestyle factors and current infection status. These may have been influenced by factors on all preceding levels.

### Data management

Low birthweight was defined as birthweight < 2.5 kg according to WHO guidelines^48^. Birth season was categorised as wet or dry based on whether the total monthly rainfall was higher or lower than the monthly median rainfall^42^. Household socioeconomic status scores had previously been calculated using principal component analysis^42^ based on variables relating to the building materials and the number of rooms or items collectively owned^46^. These scores were split into three groups: low, middle and high SES. Malaria infection below the age of five was further split into clinical and asymptomatic malaria depending on whether children presented with or without fever, respectively. In the absence of Ugandan reference values for lipid levels, the child appropriate reference values from the American Heart Association^25^ were used to categorise the lipid levels into the following three groups: (1) acceptable, (2) borderline, and (3) abnormal levels. Abnormal levels were defined as LDL ≥ 3.4 mmol/L, HDL < 1.0 mmol/L, TC ≥ 5.1 mmol/L, and triglycerides ≥ 1.47 mmol/L. Borderline levels were defined as LDL 2.8–3.3 mmol/L, HDL 1.0–1.2 mmol/L, TC 4.3–5.1 mmol/L, and triglycerides 1.0–1.46 mmol/L. Normal levels were defined as LDL < 2.8 mmol/L, HDL > 1.2 mmol/L, TC < 4.3 mmol/L, and triglycerides < 1.0 mmol/L. These categorical variables were only used to summarise the prevalence of abnormality in the cohort. For all further analyses, lipid levels were analysed as continuous outcomes.

The distribution of the outcome variables (HDL, LDL, triglycerides, and TC) were assessed using histograms and Q–Q plots. Apart from triglycerides, all outcomes were approximately normally distributed. Triglyceride levels were right skewed and were therefore log transformed (natural log), after which they approximately followed the normal distribution.

The proportion of missing data was small (0–4%), except for birthweight (17%), Tanner pubic hair stage (45%), and Tanner breast development stage in girls (49%). Since birthweight was only one of many exposure variables and not the main focus of this study, we did not use formal statistical techniques for handling missing data. The puberty stage variables were not included in the final models due to the large percentage of missing data.

### Sample size

The sample size was determined by the initial EMaBS trial objectives and for the BP study as many children as possible were included. For exposure variables with prevalence 25%, this analysis has 80% power to detect an absolute difference of 0.15 mmol/L in mean lipid level between exposed and unexposed participants.

### Statistical analysis

Data were analysed using STATA version 15 (StataCorp, Texas, United States). The arithmetic mean was calculated for HDL, LDL and TC, whereas for triglycerides the geometric mean was calculated since analyses were on the log scale. Linear regression was used to examine the associations between the exposure variables and lipid levels. For the multivariable analysis, a hierarchical modelling approach was used analogous to the method described by Victora et al.^49^. Sex and age at measurement were considered confounders a priori thus included in all models. For the first model, sex and age were included together with the variables at the most distal level (SES). The distal variables were added simultaneously irrespective of whether they were associated with the outcome in the crude analysis. Variables with a p-value < 0.10 in the adjusted model were retained and all the variables at the second level (reproductive factors) were added to the model. This process was repeated up to the most proximal level i.e. lifestyle factors and current infection. These models were independently fitted for each lipid parameter. The estimates from the linear regression models for triglycerides were back-transformed to derive the geometric mean ratio (GMR). Collinearity was assessed by comparing the standard errors from the crude models to those from the adjusted model. None of the variables was considered to be an effect modifier a priori.

## Supplementary Information

Supplementary Tables.

## Data Availability

The datasets generated and/or analysed during the current study are not publicly available because ethical approval for public sharing was not obtained, but are available from the corresponding author on reasonable request.
