# Supplementary material for: The determinants of lipid profiles in early adolescence in a Ugandan birth cohort
Source: Sci Rep. 2021 Aug 13;11:16503. doi: 10.1038/s41598-021-96035-x (PMC8363641; doi:10.1038/s41598-021-96035-x)
Supplement: Supplementary file 1 — Supplementary Tables. [file 41598_2021_96035_MOESM1_ESM.docx]

# **Supplementary Information**

**MANUSCRIPT TITLE:**

The determinants of lipid profiles in early adolescence in a Ugandan birth cohort

**AUTHORS:**

Jan Pieter R. Koopman, Swaib A. Lule, Christopher Zziwa, Hellen Akurut, Lawrence Lubyayi, Margaret Nampijja, Florence Akello, Priscilla Balungi, Josephine Tumusiime, Gloria Oduru, Alison M. Elliott, Emily L. Webb, John Bradley

# **Supplementary Tables**

## **Supplementary Table S1: Distribution of risk factors and their associations with LDL (n=1109)**

|  | **N (%)** | **Mean (95%CI)** | **Crude change in LDL for every one-unit increase of the exposure variable from baseline**  **(95%CI)** | **P value** | **Adjusted change in LDL for every one-unit increase of the exposure variable from baseline**  **(95%CI)** | **P value** |
| --- | --- | --- | --- | --- | --- | --- |
| **LEVEL 1** |  |  |  |  | **Model 1 (n=1080)** | |
| Household SES  Low  Middle  High | 141 (13)  634 (58)  319 (29) | 2.31(2.19, 2.42)  2.25 (2.20, 2.30)  2.35 (2.28, 2.42) | Reference  -0.05 (-0.17, 0.07)  0.05 (-0.09, 0.18) | 0.09 | Reference  -0.08 (-0.20, 0.05)  0.02 (-0.11, 0.16) | 0.09 |
| Maternal education  None  Primary  Senior  Tertiary | 28 (3)  537 (49)  433 (39)  109 (10) | 2.18 (1.92, 2.43)  2.26 (2.21, 2.32)  2.32 (2.26, 2.38)  2.28 (2.16, 2.41) | -0.08 (-0.33, 0.17)  Reference  0.06 (-0.03, 0.14)  0.02 (-0.11, 0.16) | 0.48 | -0.10 (-0.35, 0.16)  Reference  0.05 (-0.04, 0.13)  -0.01 (-0.14, 0.14) | 0.56 |
| Marital status  Single  Married  Other^#^ | 116 (10)  957 (87)  35 (3) | 2.25 (2.13, 2.37)  2.30 (2.26, 2.34)  2.00 (1.79, 2.22) | -0.05 (-0.17, 0.08)  Reference  -0.30(-0.52, -0.08) | 0.03 | -0.07 (-0.20, 0.06)  Reference  -0.26 (-0.50, 0.02) | 0.06 |
| Area of residence  Urban  Rural | 764 (70)  333 (30) | 2.30 (2.25, 2.35)  2.27 (2.20, 2.33) | Reference  -0.03 (-0.12, 0.05) | 0.47 | Reference  -0.02 (-0.11, 0.07) | 0.64 |
| **LEVEL 2** |  |  |  |  | **Model 2 (n=1061)** | |
| Maternal age (years)  14-19  20-24  25-29  30+ | 218 (20)  423 (38)  268 (24)  200 (18) | 2.24 (2.16, 2.33)  2.25 (2.19, 2.31)  2.36 (2.28, 2.45)  2.30 (2.20, 2.39) | Reference  0.01 (-0.10, 0.12)  0.12 (0.00, 0.24)  0.05 (-0.08, 0.18) | 0.13 | Reference  0.03 (-0.10, 0.15)  0.14 (-0.01, 0.30)  0.09 (-0.09, 0.27) | 0.23 |
| Maternal BMI (kg/m^2^) |  |  | 0.01 (0.00-0.03) | 0.01 | 0.01 (0.00, 0.02) | 0.08 |
| Parity  1  2  3  4+ | 250 (23)  291 (26)  213 (19)  355 (32) | 2.26 (2.17, 2.34)  2.29 (2.22, 2.36)  2.34 (2.26, 2.43)  2.27 (2.20, 2.34) | Reference  0.04 (-0.08, 0.15)  0.09 (-0.03, 0.21)  0.01 (-0.10, 0.12) | 0.49 | Reference  0.00 (-0.12, 0.13)  -0.01 (-0.16, 0.14)  -0.09 (-0.25, 0.06) | 0.47 |
| Alcohol during pregnancy  No  Yes | 768 (69)  340 (31) | 2.27 (2.23, 2.32)  2.31 (2.25, 2.38) | Reference  0.04 (-0.04, 0.13) | 0.35 | Reference  0.04 (-0.05, 0.13) | 0.39 |
| Maternal infection  *Sm*  No  Yes  *Ascaris*  No  Yes  *Trichuris*  No  Yes  Hookworm  No  Yes  Malaria  No  Yes | 902 (82)  200 (18)  1074 (97)  28 (3)  1006 (91)  96 (9)  658 (60)  444 (40)  983 (90)  108 (10) | 2.29 (2.25, 2.33)  2.27 (2.19, 2.35)  2.29 (2.25, 2.33)  2.32 (2.06, 2.57)  2.29 (2.25, 2.33)  2.30 (2.15, 2.46)  2.30 (2.25, 2.35)  2.27 (2.21, 2.33)  2.30 (2.26, 2.34)  2.17 (2.06, 2.28) | Reference  -0.02 (-0.12, 0.09)  Reference  0.02 (-0.22, 0.28)  Reference  0.02 (-0.12, 0.16)  Reference  -0.03 (-0.11, 0.05)  Reference  -0.13 (-0.27, 0.00) | 0.76  0.82  0.80  0.41  0.05 | Reference  0.00 (-0.11, 0.10)  Reference  0.05 (-0.21, 0.31)  Reference  0.04 (-0.10, 0.19)  Reference  -0.02 (-0.11, 0.07)  Reference  -0.11 (-0.25, 0.02) | 0.95  0.72  0.56  0.64  0.11 |
| Treatment allocation 1  Placebo  Albendazole | 561 (51)  548 (49) | 2.31 (2.25, 2.36)  2.27 (2.21, 2.32) | Reference  -0.04 (-0.11, 0.04) | 0.35 | Reference  -0.03 (-0.11, 0.05) | 0.41 |
| Treatment allocation 2  Placebo  Praziquantel | 557 (50)  552 (50) | 2.31 (2.25, 2.36)  2.27 (2.21, 2.33) | Reference  -0.04 (-0.12, 0.05) | 0.35 | Reference  -0.03 (-0.11, 0.05) | 0.43 |
| **LEVEL 3** |  |  |  |  | **Model 3 (n=890)** | |
| Sex  Female  Male | 534 (48)  575 (52) | 2.34 (2.28, 2.40)  2.24 (2.18, 2.29) | Reference  -0.11(-0.18, -0.03) | 0.008 | Reference  -0.07 (-0.16, 0.01) | 0.10 |
| Low birthweight  No  Yes | 858 (93)  65 (7) | 2.30 (2.26, 2.35)  2.25 (2.11, 2.38) | Reference  -0.06 (-0.22, 0.11) | 0.49 | Reference  -0.05 (-0.22, 0.12) | 0.58 |
| Mode of delivery  Vaginal  C-section/instrument | 996 (90)  111 (10) | 2.28 (2.24, 2.32)  2.36 (2.31, 2.49) | Reference  0.08 (-0.05, 0.21) | 0.23 | Reference  0.01 (-0.13, 0.15) | 0.92 |
| Place of delivery  Hospital  Home  Other | 816 (74)  119 (11)  173 (16) | 2.30 (2.25, 2.34)  2.18 (2.07, 2.29)  2.32 (2.22, 2.43) | Reference  -0.13 (-0.24, 0.01)  0.03 (-0.08, 0.14) | 0.16 | Reference  0.00 (-0.28, 0.28)  0.05 (-0.10, 0.20) | 0.82 |
| Season  Wet  Dry | 565 (51)  544 (49) | 2.28 (2.23, 2.34)  2.29 (2.24, 2.35) | Reference  0.01 (-0.07, 0.09) | 0.77 | Reference  -0.02 (-0.11, 0.09) | 0.66 |
| Exclusively breastfed at 6 weeks  No  Yes | 353 (32)  743 (68) | 2.28 (2.21, 2.36)  2.29 (2.25, 2.34) | Reference  0.01 (-0.07, 0.09) | 0.88 | Reference  0.00 (-0.10, 0.09) | 0.94 |
| **LEVEL 4** |  |  |  |  | **Model 4 (n=1063)** | |
| Infections below the age of 5  *Sm*  No  Yes  *Ascaris*  No  Yes  *Trichuris*  No  Yes  Hookworm  No  Yes  Asymptomatic malaria  No  Yes  Clinical malaria  No  Yes  HIV  Unexposed  Exposed, but uninfected  Infected | 1067 (97)  32 (3)  1042 (95)  57 (5)  988 (90)  111 (10)  1076 (98)  23 (2)  975 (89)  122 (11)  468 (42)  641 (58)  992 (89)  99 (9)  18 (2) | 2.29 (2.24, 2.33)  2.26 (2.08, 2.44)  2.29 (2.25, 2.33)  2.26 (2.08, 2.43)  2.28 (2.24, 2.32)  2.40 (2.25, 2.55)  2.29 (2.25, 2.33)  2.20 (1.84, 2.55)  2.39 (2.26, 2.35)  2.16 (2.04, 2.28)  2.32 (2.26, 2.38)  2.26 (2.12, 2.31)  2.30 (2.26, 2.34)  2.19 (2.08, 2.31)  2.06 (1.74, 2.39) | Reference  -0.03 (-0.26, 0.20)  Reference  -0.03 (-0.21, 0.15)  Reference  0.13 (0.00, 0.26)  Reference  -0.09 (-0.37, 0.18)  Reference  -0.15 (-0.27,-0.02)  Reference  -0.06 (-0.13, 0.02)  Reference  -0.11 (-0.25, 0.03)  -0.24 (-0.55, 0.07) | 0.81  0.73  0.05  0.51  0.02  0.17  0.10 | Reference  -0.02 (-0.26, 0.22)  Reference  -0.06 (-0.25, 012)  Reference  0.15 (0.02, 0.29)  Reference  -0.09 (-0.37, 0.18)  Reference  -0.11 (-0.24, 0.02)  -0.05 (0.13, 0.03)  Reference  -0.11 (-0.25, 0.03)  -0.16 (-0.49, 0.15) | 0.89  0.50  0.03  0.51  0.10  0.23  0.18 |
| Treatment allocation 3 ^±^  Placebo  Albendazole | 550 (50)  547 (50) | 2.29 (2.23, 2.34)  2.29 (2.23, 2.35) | Reference  0.00 (-0.08, 0.08) | 0.99 | Reference  -0.01 (-0.09, 0.07) | 0.72 |
| **LEVEL 5** |  |  |  |  | **Model 5 (n=954)** | |
| Age (years) |  |  | 0.08 (0.00, 0.15) | 0.06 | 0.07 (-0.02, 0.16) | 0.11 |
| BMI (kg/m^2^) |  |  | 0.04 (0.02, 0.06) | <0.001 | 0.03 (0.01, 0.06) | 0.006 |
| Current infections  *Sm*  No  Yes  *Trichuris*  No  Yes  Hookworm  No  Yes  Malaria  No  Yes | 957 (90)  109 (10)  1027 (96)  39 (4)  1057 (99)  9 (1)  1059 (98)  21 (2) | 2.28 (2.24, 2.33)  2.31 (2.20, 2.42)  2.29 (2.24, 2.33)  2.30 (2.12, 2.50)  2.29 (2.25, 2.33)  1.97 (1.67, 2.26)  2.30 (2.26, 2.34)  1.77 (1.50, 2.04) | Reference  0.03 (-0.11, 0.16)  Reference  0.02 (-0.19, 0.23)  Reference  -0.32 (-0.76, 0.11)  Reference  -0.53(-0.82, -0.24) | 0.70  0.84  0.15  <0.001 | Reference  0.00 (-0.14, 0.14)  Reference  0.02 (-0.22, 0.25)  Reference  -0.14 (-0.61, 0.32)  Reference  -0.51 (-0.81, -0.21) | 0.99  0.89  0.55  0.001 |
| Days of fruit eaten/week  None  1-3  4-7 | 113 (11)  616 (57)  346 (32) | 2.35 (2.24, 2.47)  2.28 (2.23, 2.33)  2.29 (2.21, 2.36) | Reference  -0.08 (-0.21, 0.06)  -0.07 (-0.21, 0.07) | 0.53 | Reference  -0.20 (-0.35, -0.05)  -0.14 (-0.30, 0.03) | 0.03 |
| Days of vegetables eaten/week  None  1-3  4-7 | 103 (9)  572 (53)  412 (38) | 2.27 (2.16, 2.38)  2.30 (2.25, 2.36)  2.30 (2.25, 2.36) | Reference  0.04 (-0.10, 0.17)  0.01 (-0.13, 0.15) | 0.77 | Reference  0.04 (-0.12, 0.18)  0.00 (-0.16, 0.15) | 0.71 |
| Days of animal protein eaten/week  None  1-3  4-7 | 119 (11)  780 (82)  191 (17) | 2.21 (2.10, 2.33)  2.37 (2.23, 2.32)  2.42 (2.31, 2.53) | Reference  0.06 (-0.06, 0.19)  0.20 (0.05, 0.35) | 0.01 | Reference  0.10 (-0.04, 0.24)  0.27 (0.11, 0.44) | 0.002 |
| Days of sugared drinks taken/week  None  1-3  4-7 | 424 (39)  487 (45)  172 (16) | 2.25 (2.19, 2.31)  2.32 (2.26, 2.38)  2.30 (2.21, 2.40) | Reference  0.07 (-0.13, 0.16)  0.06 (-0.06, 0.17) | 0.24 | Reference  0.07 (-0.02, 0.17)  0.03 (-0.09, 0.16) | 0.31 |
| PE at school  No  Yes | 384 (35)  710 (65) | 2.32 (2.25, 2.40)  2.27 (2.22, 2.31) | Reference  -0.06 (-0.14, 0.03) | 0.18 | Reference  -0.07 (-0.16, 0.01) | 0.10 |

SES: socioeconomic status; *Sm: Schistosoma mansoni;* PE: physical education # Other: widowed, divorced, or separated.

Model 1: Age, sex + other variables at level 1

Model 2: Age, sex, household SES, marital status + other variables at level 2

Model 3: Age, sex, household SES, marital status, maternal BMI + other variables at level 3

Model 4: Age, sex, household SES, marital status, maternal BMI + other variables at level 4

Model 5: Age, sex, household SES, marital status, maternal BMI, *trichuris* below age of 5, malaria below age of 5 + other variables at level 5

## **Supplementary Table S2: Distribution of risk factors and their associations with HDL (n=1096)**

|  | **N (%)** | **Mean (95%CI)** | **Crude change in HDL for every one-unit increase of the exposure variable from baseline**  **(95%CI)** | **P value** | **Adjusted change in HDL for every one-unit increase of the exposure variable from baseline**  **(95%CI)** | **P value** |
| --- | --- | --- | --- | --- | --- | --- |
| **LEVEL 1** |  |  |  |  | **Model 1 (n=1068)** | |
| Household SES  Low  Middle  High | 140 (13)  625 (58)  316 (29) | 1.06 (1.00, 1.12)  1.12 (1.10, 1.15)  1.20 (1.16, 1.24) | Reference  0.06 (0.00, 0.12)  0.14 (0.07, 0.21) | <0.001 | Reference  0.05 (-0.01, 0.12)  0.13 (0.05, 0.19) | <0.001 |
| Maternal education  None  Primary  Senior  Tertiary | 27 (2)  534 (49)  425 (39)  108 (10) | 0.94 (0.81, 1.06)  1.12 (1.09, 1.15)  1.16 (1.13, 1.19)  1.15 (1.09, 1.22) | -0.19 (-0.31, -0.06)  Reference  0.04 (-0.01, 0.08)  0.03 (-0.03, 0.10) | 0.005 | -0.17 (-0.30, -0.04)  Reference  0.03 (-0.02, 0.07)  0.02 (-0.06, 0.09) | 0.03 |
| Marital status  Single  Married  Other^#^ | 113 (10)  947 (87)  35 (3) | 1.19 (1.11, 1.27)  1.13 (1.11, 1.15)  1.09 (0.99, 1.17) | 0.06 (-0.01, 0.12)  Reference  -0.06 (-0.17, 0.06) | 0.14 | 0.05 (-0.01, 0.12)  Reference  -0.02 (-0.14, 0.11) | 0.27 |
| Area of residence  Urban  Rural | 756 (70)  329 (30) | 1.14 (1.12, 1.17)  1.12 (1.08, 1.16) | Reference  -0.02 (-0.06, 0.02) | 0.28 | Reference  -0.01 (-0.06, 0.03) | 0.51 |
| **LEVEL 2** |  |  |  |  | **Model 2 (n=1046)** | |
| Maternal age (years)  14-19  20-24  25-29  30+ | 215 (20)  420 (38)  255 (24)  195 (18) | 1.67 (1.12, 1.22)  1.11 (1.08, 1.14)  1.11 (1.07, 1.15)  1.18 (1.14, 1.24) | Reference  -0.05 (-0.11, 0.00)  -0.05 (-0.11, 0.01)  0.02 (-0.05, 0.09) | 0.03 | Reference  -0.04 (-0.11, 0.02)  -0.03 (-0.11, 0.05)  0.05 (-0.05, 0.14) | 0.04 |
| Maternal BMI (kg/m^2^) |  |  | 0.00 (-0.01, 0.01) | 0.99 | 0.00 (-0.01, 0.00) | 0.29 |
| Parity  1  2  3  4+ | 249 (23)  287 (26)  208 (19)  352 (32) | 1.16 (1.11, 1.20)  1.15 (1.11, 1.19)  1.12 (1.08, 1.17)  1.12 (1.09, 1.16) | Reference  -0.01 (-0.07, 0.05)  -0.03 (-0.09, 0.03)  -0.03 (-0.09, 0.02) | 0.60 | Reference  0.01 (-0.05, 0.08)  -0.02 (-0.10, 0.05)  -0.04 (-0.12, 0.04) | 0.45 |
| Alcohol during pregnancy  No  Yes | 758 (69)  337 (31) | 1.13 (1.11, 1.16)  1.15 (1.11, 1.18) | Reference  0.01 (-0.03, 0.06) | 0.51 | Reference  0.02 (-0.03, 0.06) | 0.46 |
| Maternal infection  *Sm*  No  Yes  *Ascaris*  No  Yes  *Trichuris*  No  Yes  Hookworm  No  Yes  Malaria  No  Yes | 892 (82)  197 (18)  1061 (97)  28 (3)  992 (91)  97 (9)  647 (59)  442 (41)  969 (90)  108 (10) | 1.13 (1.11, 1.15)  1.17 (1.12, 1.22)  1.14 (1.12, 1.16)  1.08 (0.91, 1.25)  1.14 (1.11, 1.16)  1.12 (1.05, 1.18)  1.17 (1.14, 1.19)  1.09 (1.06, 1.12)  1.14 (1.12, 1.16)  1.11 (1.05, 1.18) | Reference  0.04 (-0.01, 0.09)  Reference  -0.05 (-0.18, 0.07)  Reference  -0.02 (-0.09, 0.05)  Reference  -0.07 (-0.12, -0.03)  Reference  -0.02 (-0.09, 0.04) | 0.14  0.39  0.57  <0.001  0.47 | Reference  0.04 (-0.01, 0.09)  Reference  -0.04 (-0.17, 0.09)  Reference  0.02 (-0.06, 0.09)  Reference  -0.06 (-0.10, -0.01)  Reference  -0.02 (-0.09, 0.05) | 0.15  0.55  0.66  0.01  0.66 |
| Treatment allocation 1  Placebo  Albendazole | 553 (50)  543 (50) | 1.13 (1.10, 1.16)  1.15 (1.12, 1.17) | Reference  0.02 (-0.02, 0.06) | 0.46 | Reference  0.02 (-0.02, 0.06) | 0.30 |
| Treatment allocation 2  Placebo  Praziquantel | 551 (50)  545 (50) | 1.16 (1.13, 1.18)  1.12 (1.09, 1.15) | Reference  -0.04 (-0.08, 0.01) | 0.08 | Reference  -0.03 (-0.08, 0.00) | 0.08 |
| **LEVEL 3** |  |  |  |  | **Model 3 (n=882)** | |
| Sex  Female  Male | 527 (48)  569 (52) | 1.13 (1.11, 1.16)  1.14 (1.11, 1.17) | Reference  0.00 (-0.05, 0.04) | 0.84 | Reference  0.00 (-0.04, 0.05) | 0.88 |
| Low birthweight  No  Yes | 850 (93)  63 (7) | 1.14 (1.12, 1.17)  1.03 (0.96, 1.10) | Reference  -0.11(-0.19. -0.03) | 0.01 | Reference  -0.09 (-0.18, 0.00) | 0.05 |
| Mode of delivery  Vaginal  C-section/instrument | 986 (90)  108 (10) | 1.13 (1.11, 1.15)  1.23 (1.17, 1.29) | Reference  0.10 (0.03, 0.17) | 0.004 | Reference  0.10 (0.02, 0.17) | 0.01 |
| Place of delivery  Hospital  Home  Other | 808 (74)  115 (10)  172 (16) | 1.14 (1.12, 1.17)  1.08 (1.01, 1.14)  1.15 (1.10, 1.21) | Reference  -0.07 (-0.13, 0.00)  0.01 (-0.05, 0.06) | 0.12 | Reference  -0.05 (-0.20, 0.09)  -0.06 (-0.13, 0.02) | 0.24 |
| Season  Wet  Dry | 558 (51)  538 (49) | 1.16 (1.13, 1.19)  1.11 (1.09, 1.14) | Reference  -0.04 (-0.08, 0.00) | 0.03 | Reference  -0.06 (-0.11, -0.02) | 0.007 |
| Exclusively breastfed at 6 weeks  No  Yes | 346 (32)  737 (68) | 1.14 (1.10, 1.17)  1.14 (1.11, 1.16) | Reference  0.00 (-0.04, 0.05) | 0.86 | Reference  0.03 (-0.02, 0.08) | 0.25 |
| **LEVEL 4** |  |  |  |  | **Model 4 (n=875)** | |
| Infections below the age of 5  *Sm*  No  Yes  *Ascaris*  No  Yes  *Trichuris*  No  Yes  Hookworm  No  Yes  Asymptomatic malaria  No  Yes  Clinical malaria  No  Yes  HIV  Unexposed  Exposed, but uninfected  Infected | 1053 (97)  33 (3)  1029 (95)  57 (5)  977 (90)  109 (10)  1063 (98)  23 (2)  962 (89)  122 (11)  640 (42)  636 (58)  980 (89)  98 (9)  18 (2) | 1.14 (1.12, 1.16)  1.12 (1.00, 1.24)  1.14 (1.12, 1.16)  1.04 (0.96, 1.13  1.14 (1.12, 1.17)  1.10 (1.04, 1.16)  1.14 (1.12, 1.16)  1.05 (0.91, 1.18)  1.15 (1.13, 1.17)  1.06 (0.99, 1.11)  1.17 (1.14, 1.20)  1.12 (1.10, 1.14)  1.14 (1.12, 1.16)  1.15 (1.08, 1.22)  1.00 (0.82, 1.18) | Reference  -0.02 (-0.13, 0.10)  Reference  -0.10(-0.19, -0.01)  Reference  -0.04 (-0.11, 0.02)  Reference  -0.09 (-0.23, 0.05)  Reference  -0.09(-0.16, -0.03)  Reference  -0.05 (-0.09, -0.01)  Reference  0.01 (-0.06, 0.08)  -0.14 (-0.30, 0.02) | 0.79  0.03  0.21  0.21  0.005  0.01  0.21 | Reference  -0.04 (-0.18, 0.10)  Reference  -0.06 (-0.16, 0.05)  Reference  0.01 (-0.07, 0.08)  Reference  -0.07 (-0.22, 0,08)  Reference  -0.08 (-0.15, 0.00)  Reference  -0.03 (-0.07, 0.02)  Reference  0.03 (-0.04, 0.11)  -0.06 (-0.22, 0.10) | 0.55  0.28  0.88  0.37  0.04  0.25  0.53 |
| Treatment allocation 3  Placebo  Albendazole | 542 (50)  542 (50) | 1.14 (1.11, 1.17)  1.13 (1.10, 1.16) | Reference  -0.01 (-0.05, 0.03) | 0.57 | Reference  -0.02 (-0.06, 0.03) | 0.48 |
| **LEVEL 5** |  |  |  |  | **Model 5 (n=875)** | |
| Age (years) |  |  | -0.01 (-0.05, 0.03) | 0.48 | -0.02 (-0.07, 0.03) | 0.39 |
| BMI (kg/m^2^) |  |  | 0.00 (-0.01, 0.01) | 0.74 | 0.00 (-0.02, 0.01) | 0.64 |
| Current infections  *Sm*  No  Yes  *Trichuris*  No  Yes  Hookworm  No  Yes  Malaria  No  Yes | 944 (90)  109 (10)  1014 (96)  39 (4)  1044 (99)  9 (1)  1045 (98)  21 (2) | 1.14 (1.12, 1.16)  1.09 (1.03, 1.15)  1.14 (1.11, 1.16)  1.10 (0.96, 1.24)  1.14 (1.11,1.16)  0.97 (0.71, 1.22)  1.15 (1.13, 1.17)  0.65 (0.50, 0.81) | Reference  -0.05 (-0.11, 0.02)  Reference  -0.04 (-0.15, 0.07)  Reference  -0.17 (-0.40, 0.05)  Reference  -0.49(-0.64, -0.35) | 0.17  0.51  0.13  <0.001 | Reference  -0.04 (-0.12, 0.04)  Reference  0.00 (-0.13, 0.13)  Reference  -0.07 (-0.32, 0.18)  Reference  -0.40 (-0.56, -0.23) | 0.37  0.98  0.59  <0.001 |
| Days of fruit eaten/week  None  1-3  4-7 | 111 (10)  610 (58)  340 (32) | 1.11 (1.05, 1.17)  1.15 (1.12, 1.18)  1.12 (1.09, 1.16) | Reference  0.04 (-0.03, 0.11)  0.01 (-0.06, 0.09) | 0.34 | Reference  -0.01 (-0.10, 0.07)  -0.02 (-0.11, 0.07) | 0.85 |
| Days of vegetables eaten/week  None  1-3  4-7 | 102 (10)  563 (52)  408 (38) | 1.14 (1.07, 1.21)  1.14 (1.11, 1.17)  1.14 (1.11, 1.17) | Reference  0.00 (-0.07, 0.07)  0.00 (-0.07, 0.07) | 0.98 | Reference  -0.09 (-0.18, 0.00)  -0.09 (-0.18, 0.04) | 0.09 |
| Days of animal protein eaten/week  None  1-3  4-7 | 118 (11)  769 (82)  190 (18) | 1.06 (1.00, 1.13)  1.14 (1.12, 1.17)  1.17 (1.12, 1.22) | Reference  0.08 (0.01, 0.14)  0.10 (0.03, 0.18) | 0.03 | Reference  0.05 (-0.03, 0.12)  0.10 (0.01, 0.19) | 0.10 |
| Days of sugared drinks taken/week  None  1-3  4-7 | 419 (39)  478 (45)  173 (16) | 1.11 (1.08, 1.14)  1.13 (1.10, 1.16)  1.24 (1.19, 1.29) | Reference  0.02 (-0.03, 0.06)  0.13 (0.07, 0.19) | <0.001 | Reference  0.01 (-0.04, 0.07)  0.13 (0.06, 0.20) | <0.001 |
| PE at school  No  Yes | 382 (35)  699 (65) | 1.13 (1.10, 1.16)  1.14 (1.12, 1.17) | Reference  0.01 (-0.03, 0.06) | 0.51 | Reference  0.00 (-0.05, 0.04) | 0.88 |

SES: socioeconomic status; *Sm: Schistosoma mansoni;* PE: physical education # Other: widowed, divorced, or separated.

Model 1: Age, sex + other variables at level 1

Model 2: Age, sex, household SES, maternal education + other variables at level 2

Model 3: Age, sex, household SES, maternal education, maternal age, maternal hookworm infection, treatment allocation to praziquantel + other variables at level 3

Model 4: Age, sex, household SES, maternal education, maternal age, maternal hookworm infection, treatment allocation to praziquantel, low birthweight, mode of delivery, season + other variables at level 4

Model 5: Age, sex, household SES, maternal education, maternal age, maternal hookworm infection, treatment allocation to praziquantel, low birthweight, mode of delivery, season, malaria infection below the age of 5 + other variables at level 5

## **Supplementary Table S3: Distribution of risk factors and their associations with total cholesterol (n=1113)**

|  | **N (%)** | **Mean (95%CI)** | **Crude change in TC for every one-unit increase of the exposure variable from baseline**  **(95%CI)** | **P value** | **Adjusted change in TC for every one-unit increase of the exposure variable from baseline**  **(95%CI)** | **P value** |
| --- | --- | --- | --- | --- | --- | --- |
| **LEVEL 1** |  |  |  |  | **Model 1 (n=1098)** | |
| Household SES  Low  Middle  High | 141 (13)  637 (58)  320 (29) | 3.79 (3.65, 3.92)  3.78 (3.71, 3.84)  3.93 (3.85, 4.01) | Reference  -0.01 (-0.15, 0.13)  0.14 (-0.01, 0.30) | 0.02 | Reference  -0.04 (-0.19, 0.11)  0.10 (-0.05, 0.26) | 0.03 |
| Maternal education  None  Primary  Senior  Tertiary | 28 (2)  540 (49)  434 (39)  109 (10) | 3.55 (3.22, 3.88)  3.78 (3.71, 3.85)  3.87 (3.80, 3.94)  3.86 (3.71, 4.00) | -0.23 (-0.53, 0.07)  Reference  0.09 (-0.01, 0.20)  0.07 (-0.09, 0.24) | 0.08 | -0.23 (-0.53, 0.07)  Reference  0.07 (-0.03, 0.17)  0.02 (-0.14, 0.19) | 0.18 |
| Marital status  Single  Married  Other^#^ | 116 (11)  961 (86)  35 (3) | 3.84 (3.71, 3.97)  3.83 (3.78, 3.88)  3.44 (3.22, 3.67) | - 1. (-0.14, 0.16)   Reference  -0.39 (-0.65, -0.12) | 0.02 | -0.01 (-0.16, 0.14)  Reference  -0.32 (-0.61, -0.04) | 0.08 |
| Area of residence  Urban  Rural | 767 (70)  335 (30) | 3.84 (3.79, 3.90)  3.77 (3.70, 3.85) | Reference  -0.07 (-0.17, 0.03) | 0.17 | Reference  -0.05 (-0.15, 0.05) | 0.32 |
| **LEVEL 2** |  |  |  |  | **Model 2 (n=1047)** | |
| Maternal age (years)  14-19  20-24  25-29  30+ | 218 (20)  426 (38)  269 (24)  200 (18) | 3.80 (3.71, 3.90)  3.76 (3.69, 3.84)  3.91 (3.81, 4.01)  3.83 (3.73, 3.94) | Reference  -0.04 (-0.17, 0.09)  0.11 (-0.03, 0.25)  0.03 (-0.12, 0.18) | 0.12 | Reference  0.01 (-0.13, 0.16)  0.19 (0.01, 0.37)  0.14 (-0.07, 0.35) | 0.08 |
| Maternal BMI (kg/m^2^) |  |  | 0.01 (0.00, 0.03) | 0.04 | 0.01 (0.00, 0.02) | 0.19 |
| Parity  1  2  3  4+ | 252 (23)  291 (26)  213 (19)  357 (32) | 3.84 (3.72, 3.95)  3.83 (3.74, 3.91)  3.85 (3.74, 3.95)  3.79 (3.71, 3.86) | Reference  -0.01 (-0.14, 0.12)  0.01 (-0.13, 0.15)  -0.05 (-0.18, 0.08) | 0.79 | Reference  -0.04 (-0.19, 0.11)  -0.11 (-0.28, 0.06)  -0.19 (-0.37, -0.01) | 0.19 |
| Alcohol during pregnancy  No  Yes | 772 (69)  340 (31) | 3.81 (3.76, 3.87)  3.83 (3.75, 3.91) | Reference  0.01 (-0.09, 0.11) | 0.78 | Reference  0.03 (-0.08, 0.13) | 0.63 |
| Maternal infection  *Sm*  No  Yes  *Ascaris*  No  Yes  *Trichuris*  No  Yes  Hookworm  No  Yes  Malaria  No  Yes | 905 (82)  201 (18)  1078 (97)  28 (3)  1009 (91)  97 (9)  661 (60)  445 (40)  986 (90)  108 (10) | 3.82 (3.77, 3.87)  3.81 (3.72, 3.90)  3.82 (3.77, 3.87)  3.79 (3.47, 4.11)  3.82 (3.77, 3.87)  3.82 (3.65, 3.40)  3.85 (3.79, 3.90)  3.78 (3.70, 3.86)  3.83 (3.78, 3.88)  3.67 (3.52, 3.82) | Reference  -0.01 (-0.13, 0.11)  Reference  -0.03 (-0.32, 0.27)  Reference  0.00 (-0.16, 0.17)  Reference  -0.07 (-0.16, 0.03)  Reference  -0.16 (-0.31, 0.00) | 0.82  0.86  0.96  0.17  0.05 | Reference  0.00 (-0.13, 0.12)  Reference  -0.01 (-0.31, 0.30)  Reference  0.04 (-0.13, 0.21)  Reference  -0.04 (-0.15, 0.06)  Reference  -0.15 (-0.31, -0.02) | 0.97  0.97  0.65  0.39  0.08 |
| Treatment allocation 1  Placebo  Albendazole | 562 (50)  551 (50) | 3.84 (3.77, 3.91)  3.80 (3.73, 3.86) | Reference  -0.05 (-0.14, 0.05) | 0.33 | Reference  -0.05 (-0.14, 0.05) | 0.32 |
| Treatment allocation 2  Placebo  Praziquantel | 560 (50)  553 (50) | 3.85 (3.79, 3.91)  3.79 (3.71, 3.86) | Reference  -0.07 (-0.16, 0.03) | 0.16 | Reference  -0.06 (-0.15, 0.04) | 0.23 |
| **LEVEL 3** |  |  |  |  | **Model 3 (n=885)** | |
| Sex  Female  Male | 536 (48)  577 (52) | 3.89 (3.82, 3.95)  3.76 (3.69, 3.82) | Reference  -0.13 (-0.22, 0.04) | 0.005 | Reference  -0.09 (-0.20, 0.01) | 0.08 |
| Low birthweight  No  Yes | 862 (93)  65 (7) | 3.84 (3.79, 3.90)  3.72 (3.58, 3.87) | Reference  -0.11 (-0.32, 0.08) | 0.24 | Reference  -0.07 (-0.27, 0.14) | 0.52 |
| Mode of delivery  Vaginal  C-section/instrument | 999 (90)  112 (10) | 3.81 (3.76, 3.86)  3.92 (3.76, 4.08) | Reference  0.11 (-0.04, 0.26) | 0.16 | Reference  0.10 (-0.07, 0.29) | 0.24 |
| Place of delivery  Hospital  Home  Other | 819 (74)  119 (11)  174 (15) | 3.83 (3.78, 3.89)  3.63 (3.40, 3.76)  3.90 (3.79, 4.00) | Reference  -0.20(-0.35, -0.05)  0.06 (-0.06, 0.19) | 0.01 | Reference  -0.11 (-0.44, 0.22)  0.05 (-0.13, 0.22) | 0.69 |
| Season  Wet  Dry | 568 (51)  545 (49) | 3.85 (3.78, 3.91)  3.79 (3.73, 3.86) | Reference  -0.06 (-0.15, 0.04) | 0.24 | Reference  -0.11 (-0.21, 0.00) | 0.04 |
| Exclusively breastfed at 6 weeks  No  Yes | 356 (32)  744 (68) | 3.80 (3.72, 3.88)  3.83 (3.78, 3.89) | Reference  0.04 (-0.06, 0.14) | 0.47 | Reference  0.06 (-0.05, 0.18) | 0.30 |
| **LEVEL 4** |  |  |  |  | **Model 4 (n=885)** | |
| Infections below the age of 5  *Sm*  No  Yes  *Ascaris*  No  Yes  *Trichuris*  No  Yes  Hookworm  No  Yes  Asymptomatic malaria  No  Yes  Clinical malaria  No  Yes  HIV  Unexposed  Exposed, but uninfected  Infected | 1070 (97)  33 (3)  1046 (95)  57 (5)  992 (90)  111 (10)  1079 (98)  24 (2)  978 (89)  123 (11)  471 (42)  642 (58)  995 (89)  100 (9)  18 (2) | 3.82 (3.77, 3.86)  3.98 (3.46, 4.50)  3.82 (3.78, 3.87)  3.73 (3.52, 3.94)  3.81 (3.77, 3.86)  3.88 (3.72, 4.03)  3.83 (3.78, 3.87)  3.58 (3.21, 3.94)  3.84 (3.79, 3.89)  3.67 (3.53, 3.80)  3.88 (3.80, 3.95)  3.78 (3.72, 3.83)  3.83 (3.78, 3.88)  3.75 (3.61, 3.89)  3.5 (3.12, 3.88) | Reference  0.16 (-0.11, 0.43)  Reference  -0.09 (-0.30, 0.11)  Reference  0.06 (-0.09, 0.21)  Reference  -0.25 (-0.57, 0.07)  Reference  -0.17(-0.32, -0.02)  Reference  -0.10 (-0.19, 0.00)  Reference  -0.08 (-0.24, 0.08)  -0.33 (-0.70, 0.03) | 0.24  0.36  0.42  0.12  0.02  0.04  0.13 | Reference  0.21 (-0.07, 0.50)  Reference  -0.09 (-0.31, 0.13)  Reference  0.10 (-0.06, 0.27)  Reference  -0.24 (-0.56, 0.08)  Reference  -0.14 (-0.30, 0.01)  Reference  -0.05 (-0.16, 0.04)  Reference  -0.07 (-0.24, 0.10)  -0.20 (-0.58, 0.18) | 0.13  0.39  0.20  0.14  0.07  0.27  0.42 |
| Treatment allocation 3 ^±^  Placebo  Albendazole | 551 (50)  550 (50) | 3.81 (3.75, 3.87)  3.83 (3.76, 3.90) | Reference  0.02 (-0.07, 0.11) | 0.66 | 0.01 (-0.09, 0.10) | 0.85 |
| **LEVEL 5** |  |  |  |  | **Model 5 (n=952)** | |
| Age (years) |  |  | 0.04 (-0.05, 0.14) | 0.35 | 0.01 (-0.09, 0.11) | 0.86 |
| BMI (kg/m^2^) |  |  | 0.05 (0.03, 0.08) | <0.001 | 0.05 (0.02, 0.08) | 0.001 |
| Current infections  *Sm*  No  Yes  *Trichuris*  No  Yes  Hookworm  No  Yes  Malaria  No  Yes | 960 (90)  110 (10)  1031 (96)  39 (4)  1060 (99)  10 (1)  1062 (98)  21 (2) | 3.82 (3.77, 3.87)  3.76 (3.64, 3.88)  3.82 (3.77, 3.87)  3.74 (3.50, 3.99)  3.82 (3.77, 3.87)  3.44 (3.04, 3.85)  3.83 (3.78, 3.88)  3.13 (2.86, 3.40) | Reference  -0.06 (-0.22, 0.09)  Reference  -0.08 (-0.32, 0.18)  Reference  -0.38 (-0.87, 0.11)  Reference  -0.70(-1.04, -0.37) | 0.43  0.56  0.13  <0.001 | Reference  -0.05 (-0.22, 0.12)  Reference  -0.03 (-0.31, 0.25)  Reference  -0.14 (-0.67, 0.38)  Reference  -0.62 (-0.97, -0.27) | 0.56  0.83  0.60  <0.001 |
| Days of fruit eaten/week  None  1-3  4-7 | 113 (11)  618 (57)  347 (32) | 3.90 (3.76, 4.03)  3.82 (3.76, 3.89)  3.79 (3.70, 3.87) | Reference  -0.07 (-0.23, 0.08)  -0.11 (-0.28, 0.06) | 0.43 | Reference  -0.21 (-0.39, 0.03)  -0.19 (-0.38, 0.00) | 0.07 |
| Days of vegetables eaten/week  None  1-3  4-7 | 104 (9)  573 (53)  413 (38) | 3.81 (3.69, 3.93)  3.83 (3.72, 3.89)  3.80 (3.72, 3.89) | Reference   - 1. (-0.14, 0.19)   0.00 (-0.17, 0.17) | 0.88 | Reference  -0.02 (-0.20, 0.16)  -0.03 (-0.22, 0.15) | 0.91 |
| Days of animal protein eaten/week  None  1-3  4-7 | 121 (11)  782 (72)  191 (17) | 3.72 (3.59, 3.86)  3.80 (3.75, 3.86)  3.95 (3.82, 4.07) | Reference  0.08 (-0.07, 0.23)  0.23 (0.05, 0.41) | 0.02 | Reference  0.11 (-0.06, 0.27)  0.33 (0.13, 0.53) | 0.001 |
| Days of sugared drinks taken/week  None  1-3  4-7 | 425 (39)  488 (45)  174 (16) | 3.76 (3.69, 3.83)  3.82 (3.76, 3.89)  3.96 (3.82, 3.10) | Reference  0.07 (-0.04, 0.17)  0.20 (0.07, 0.34) | 0.02 | Reference  0.05 (-0.06, 0.16)  0.16 (0.00, 0.31) | 0.13 |
| PE at school  No  Yes | 385 (35)  713 (65) | 3.86 (3.78, 3.94)  3.80 (3.74, 3.85) | Reference  -0.07 (-0.16, 0.03) | 0.19 | Reference  -0.08 (-0.18, 0.02) | 0.13 |

SES: socioeconomic status; *Sm: Schistosoma mansoni;* PE: physical education # Other: widowed, divorced, or separated.

Model 1: Age, sex + other variables at level 1

Model 2: Age, sex, household SES, marital status + other variables at level 2

Model 3: Age, sex, household SES, marital status, maternal age, maternal malaria infection+ other variables at level 3

Model 4: Age, sex, household SES, marital status, maternal age, maternal malaria infection, season + other variables at level 4

Model 5: Age, sex, household SES, marital status, maternal age, maternal malaria infection, season, malaria infection below the age of 5 + other variables at level 5

## **Supplementary Table S4: Distribution of risk factors and their associations with triglyceride levels (n=1113)**

|  | **N (%)** | **Mean (95%CI)** | **Crude geometric mean ratio (95%CI** | **P value** | **Adjusted geometric mean ratio (95% CI)** | **P value** |
| --- | --- | --- | --- | --- | --- | --- |
| **LEVEL 1** |  |  |  |  | **Model 1 (n=1085)** | |
| Household SES  Low  Middle  High | 141 (13)  637 (58)  320 (29) | 0.91 (0.85, 0.98)  0.93 (0.90, 0.96)  0.90 (0.86, 0.94) | Reference  1.02 (0.94, 1.10)  0.98 (0.90, 1.07) | 0.53 | Reference  1.01 (0.93, 1.10)  0.98 (0.90, 1.07) | 0.62 |
| Maternal education  None  Primary  Senior  Tertiary | 28 (3)  540 (49)  434 (39)  109 (10) | 0.92 (0.81, 1.05)  0.89 (0.86, 0.93)  0.93 (0.89, 0.96)  0.94 (0.87, 1.03) | 1.03 (0.87, 1.22)  Reference  1.04 (0.98, 1.09)  1.05 (0.96, 1.15) | 0.53 | 1.02 (0.87, 1.21)  Reference  1.03 (0.98, 1.09)  1.05 (0.96, 1.15) | 0.63 |
| Marital status  Single  Married  Other^#^ | 116 (11)  961 (86)  35 (3) | 0.90 (0.83, 0.98)  0.92 (0.89, 0.94)  0.89 (0.75, 1.05) | 0.98 (0.90, 1.07)  Reference  0.97 (0.84, 1.13) | 0.87 | 1.00 (0.91, 1.09)  Reference  0.97 (0.83, 1.14) | 0.93 |
| Area of residence  Urban  Rural | 767 (70)  335 (30) | 0.91 (0.88, 0.94)  0.92 (0.87, 0.96) | Reference  1.01 (0.95, 1.07) | 0.74 | Reference  1.02 (0.96, 1.08) | 0.51 |
| **LEVEL 2** |  |  |  |  | **Model 2 (n=1077)** | |
| Maternal age (years)  14-19  20-24  25-29  30+ | 218 (20)  426 (38)  269 (24)  200 (18) | 0.88 (0.84, 0.94)  0.92 (0.88, 0.96)  0.98 (0.93, 1.03)  0.86 (0.81, 0.91) | Reference  1.03 (0.98, 1.11)  1.10 (1.02, 1.19)  0.97 (0.89, 1.05) | 0.08 | Reference  1.04 (0.96, 1.12)  1.10 (1.00, 1.22)  0.97 (0.86, 1.09) | 0.01 |
| Maternal BMI (kg/m^2^) |  |  | 0.00 (-0.01, 0.01) | 0.68 | 1.00 (1.00, 1.01) | 0.36 |
| Parity  1  2  3  4+ | 252 (23)  291 (26)  213 (19)  357 (32) | 0.91 (0.86, 0.96)  0.90 (0.86, 0.95)  0.95 (0.88, 0.98)  0.92 (0.87, 0.96) | Reference  0.99 (0.92, 1.07)  1.02 (0.94, 1.11)  1.01 (0.94, 1.08) | 0.92 | Reference  0.97 (0.90, 1.05)  0.99 (0.90, 1.08)  1.00 (0.90, 1.10) | 0.90 |
| Alcohol during pregnancy  No  Yes | 772 (69)  340 (31) | 0.92 (0.89, 0.95)  0.91 (0.87, 0.95) | Reference  0.99 (0.94, 1.05) | 0.75 | Reference  1.00 (0.94, 1.06) | 0.96 |
| Maternal infection  *Sm*  No  Yes  *Ascaris*  No  Yes  *Trichuris*  No  Yes  Hookworm  No  Yes  Malaria  No  Yes | 905 (82)  201 (18)  1078 (97)  28 (3)  1009 (91)  97 (9)  661 (60)  445 (40)  986 (90)  108 (10) | 0.92 (0.90, 0.95)  0.87 (0.82, 0.92)  0.92 (0.89, 0.94)  0.85 (0.77, 0.95)  0.92 (0.89, 0.94)  0.89 (0.81, 0.97)  0.90 (0.87, 0.93)  0.94 (0.90, 0.98)  0.91 (0.89, 0.94)  0.91 (0.85, 0.98) | Reference  0.94 (0.88, 1.01)  Reference  0.93 (0.79, 1.10)  Reference  0.97 (0.89, 1.06)  Reference  1.05 (1.00, 1.11)  Reference  1.00 (0.92, 1.09) | 0.07  0.39  0.53  0.07  0.98 | Reference  0.95 (0.89, 1.02)  Reference  0.94 (0.80, 1.11)  Reference  0.96 (0.87, 1.05)  Reference  1.05 (0.99, 1.11)  Reference  1.00 (0.91, 1.09) | 0.17  0.49  0.35  0.08  0.98 |
| Treatment allocation 1  Placebo  Albendazole | 562 (50)  551 (50) | 0.92 (0.88, 0.95)  0.91 (0.88, 0.94) | Reference  0.99 (0.94, 1.04) | 0.73 | Reference  0.98 (0.93, 1.03) | 0.51 |
| Treatment allocation 2  Placebo  Praziquantel | 560 (50)  553 (50) | 0.92 (0.89, 0.96)  0.90 (0.87, 0.93) | Reference  0.98 (0.93, 1.03) | 0.33 | Reference  0.97 (0.92, 1.03) | 0.33 |
| **LEVEL 3** |  |  |  |  | **Model 3 (n=912)** | |
| Sex  Female  Male | 536 (48)  577 (52) | 0.94 (0.90, 0.97)  0.89 (0.86, 0.93) | Reference  1.05 (1.00, 1.10) | 0.07 | Reference  1.02 (0.96, 1.08) | 0.51 |
| Low birthweight  No  Yes | 862 (93)  65 (7) | 0.90 (0.88, 0.93)  1.00 (0.91, 1.11) | Reference  1.11 (1.00, 1.24) | 0.07 | Reference  1.12 (1.00, 1.25) | 0.05 |
| Mode of delivery  Vaginal  C-section/instrument | 999 (90)  112 (10) | 0.92 (0.90, 0.95)  0.85 (0.78, 0.91) | Reference  0.92 (0.84, 1.00) | 0.05 | Reference  0.94 (0.86, 1.03) | 0.19 |
| Place of delivery  Hospital  Home  Other | 819 (74)  119 (11)  174 (16) | 0.90 (0.88, 0.93)  0.91 (0.84, 0.98)  0.98 (0.91, 1.05) | Reference  1.01 (0.93, 1.10)  1.08 (1.01, 1.16) | 0.09 | Reference  0.91 (0.76, 1.08)  1.10 (1.00, 1.21) | 0.06 |
| Season  Wet  Dry | 568 (51)  545 (49) | 0.91 (0.87, 0.94)  0.92 (0.89, 0.95) | Reference  1.01 (0.96, 1.07) | 0.59 | Reference  1.01 (0.96, 1.07) | 0.43 |
| Exclusively breastfed at 6 weeks  No  Yes | 356 (32)  744 (68) | 0.90 (0.86, 0.94)  0.92 (0.89, 0.95) | Reference  1.02 (0.97, 1.08) | 0.46 | Reference  1.02 (0.96, 1.09) | 0.52 |
| **LEVEL 4** |  |  |  |  | **Model 4 (n=905)** | |
| Infections below the age of 5  *Sm*  No  Yes  *Ascaris*  No  Yes  *Trichuris*  No  Yes  Hookworm  No  Yes  Asymptomatic malaria  No  Yes  Clinical malaria  No  Yes  HIV  Unexposed  Exposed, but uninfected  Infected | 1070 (3)  33 (97)  1046 (95)  57 (5)  992 (90)  111 (10)  1079 (98)  24 (2)  978 (89)  123 (11)  471 (42)  642 (58)  995 (89)  100 (9)  18 (2) | 0.91 (0.89, 0.94)  0.91 (0.77, 1.08)  0.91 (0.89, 0.94)  0.94 (0.84, 1.04)  0.91 (0.89, 0.94)  0.92 (0.86, 0.99)  0.91 (0.89, 0.94)  0.91 (0.78, 1.05)  0.91 (0.88, 0.93)  0.95 (0.88, 1.03)  0.91 (0.87, 0,95)  0.91 (0.89, 0.95)  0.91 (0.89, 0.94)  0.91 (0.84, 1.00)  0.99 (0.85, 1.15) | Reference  1.00 (0.86, 1.16)  Reference  1.03 (0.91, 1.15)  Reference  1.01 (0.92, 1.10)  Reference  0.99 (0.83, 1.18)  Reference  1.05 (0.97, 1.14)  Reference  1.01 (0.96, 1.06)  Reference  1.00 (0.92, 1.10)  1.08 (0.88, 1.33) | 0.99  0.67  0.90  0.93  0.25  0.78  0.74 | Reference  1.04 (0.87, 1.24)  Reference  1.00 (0.87, 1.15)  Reference  0.99 (0.89, 1.09)  Reference  1.01 (0.83, 1.23)  Reference  1.03 (0.94, 1.14)  Reference  0.98 (0.93, 1.04)  Reference  1.01 (0.91, 1.12)  1.03 (0.84, 1.28) | 0.69  0.99  0.81  0.90  0.45  0.58  0.93 |
| Treatment allocation 3  Placebo  Albendazole | 551 (50)  550 (50) | 0.89 (0.86, 0.92)  0.93 (0.90, 0.97) | Reference  1.05 (1.00, 1.12) | 0.08 | Reference  1.06 (1.00, 1.12) | 0.05 |
| **LEVEL 5** |  |  |  |  | **Model 5 (n=807)** | |
| Age (years) |  |  | 1.04 (1.00, 1.10) | 0.11 | 0.98 (0.92, 1.04) | 0.47 |
| BMI (kg/m^2^) |  |  | 1.02 (1.01, 1.04) | <0.001 | 1.03 (1.01, 1.05) | <0.001 |
| Current infections  *Sm*  No  Yes  *Trichuris*  No  Yes  Hookworm  No  Yes  Malaria  No  Yes | 960 (90)  110 (10)  1031 (96)  39 (4)  1060 (99)  10 (1)  1062 (98)  21 (2) | 0.92 (0.90, 0.95)  0.89 (0.83, 0.96)  0.92 (0.90, 0.95)  0.84 (0.75, 0.95)  0.92 (0.90, 0.94)  0.80 (0.61. 1.04)  0.91 (0.89, 0.93)  1.35 (1.17, 1.56) | Reference  0.97 (0.90, 1.06)  Reference  0.91 (0.79, 1.05)  Reference  0.87 (0.66, 1.14)  Reference  1.49 (1.24, 1.80) | 0.49  0.21  0.30  <0.001 | Reference  0.97 (0.88, 1.08)  Reference  0.86 (0.73, 1.03)  Reference  0.77 (0.57, 1.05)  Reference  1.47 (1.18, 1.84) | 0.58  0.10  0.10  0.001 |
| Days of fruit eaten/week  None  1-3  4-7 | 113 (11)  618 (57)  347 (32) | 0.96 (0.89, 1.04)  0.89 (0.86, 0.92)  0.94 (0.90, 0.98) | Reference  0.92 (0.85, 1.01)  0.98 (0.89, 1.07) | 0.06 | Reference  0.97 (0.87, 1.08)  1.04 (0.92, 1.16) | 0.12 |
| Days of vegetables eaten/week  None  1-3  4-7 | 104 (9)  573 (53)  412 (38) | 0.94 (0.86, 1.01)  0.91 (0.88, 0.94)  0.91 (0.87, 0.95) | Reference  0.97 (0.89, 1.07)  0.97 (0.89, 1.07) | 0.83 | Reference  1.00 (0.89, 1.11)  1.00 (0.88, 1.12) | 0.97 |
| Days of animal protein eaten/week  None  1-3  4-7 | 121 (11)  782 (82)  191 (17) | 0.94 (0.88, 1.02)  0.91 (0.88, 0.93)  0.91 (0.86, 0.97) | Reference  0.96 (0.89, 1.05)  0.97 (0.87, 1.07) | 0.69 | Reference  1.00 0.90, 1.10)  1.02 (0.91, 0.15) | 0.83 |
| Days of sugared drinks taken/week  None  1-3  4-7 | 425 (39)  488 (45)  174 (16) | 0.92 (0.88, 0.96)  0.92 (0.88, 0.95)  0.89 (0.83, 0.95) | Reference  1.00 (0.94, 1.06)  0.97 (0.90, 1.05) | 0.72 | Reference  0.99 (0.92, 1.06)  0.94 (0.86, 1.03) | 0.46 |
| PE at school  No  Yes | 385 (35)  713 (65) | 0.94 (0.91, 0.98)  0.90 (0.87, 0.93) | Reference  0.95 (0.90, 1.01) | 0.08 | Reference  0.96 (0.90, 1.03) | 0.24 |

SES: socioeconomic status; *Sm: Schistosoma mansoni;* PE: physical education # Other: widowed, divorced, or separated.

Model 1: Age, sex + other variables at level 1

Model 2: Age, sex + other variables at level 2

Model 3: Age, sex, maternal age, maternal hookworm infection + other variables at level 3

Model 4: Age, sex, maternal age, maternal hookworm infection, low birthweight, place of delivery + other variables at level 4

Model 5: Age, sex, maternal age, maternal hookworm infection, low birthweight, place of delivery, treatment allocation to albendazole (child) + other variables at level 5
